# Supplementary material for: The Effect of Conjugation of Ciprofloxacin and Moxifloxacin with Fatty Acids on Their Antibacterial and Anticancer Activity
Source: Int J Mol Sci. 2022 Jun 2;23(11):6261. doi: 10.3390/ijms23116261 (PMC9181188; doi:10.3390/ijms23116261)
Supplement: Supplementary file 1 [file ijms-23-06261-s001.zip › ijms-1737141-supplementary.pdf]

Supporting Information

The effect of conjugation of ciprofloxacin and moxifloxacin with fatty acids on their antibacterial and anticancer activity

Alicja Chrzanowska[1], Marta Struga[1], Piotr Roszkowski[2,*], Michał Koliński[3], Sebastian Kmiecik[4], Karolina Jałbrzykowska[1], Anna Zabost[5], Joanna Stefańska[6], Ewa Augustynowicz-Kopeć[5], Małgorzata Wrzosek[7], Anna Bielenica[1,*]

[1] Chair and Department of Biochemistry, Medical University of Warsaw, ul. Banacha 1, 02-097 Warsaw, Poland; achrzanowska@wum.edu.pl (A.Ch); mstruga@wum.edu.pl (M.S), kjalbrzykowska@wum.edu.pl (K.J.), abielenica@wum.edu,pl (A.B)

[2] Faculty of Chemistry, University of Warsaw, Pasteura 1, 02-093 Warszawa, Poland: roszkowski@chem.uw.edu.pl (P.R)

[3] Bioinformatics Laboratory, Mossakowski Medical Research Institute, Polish Academy of Sciences, 5 Pawinskiego St., 02-106 Warsaw, Poland; kolinski.michal@gmail.com (M.K.)

[4] Biological and chemical Research Centre, Faculty of Chemistry, University of Warsaw, 02-089 Warsaw, Poland; sebastian.kmiecik@gmail.com (S.K.)

[5] Department of Microbiology, National Tuberculosis and Lung Diseases Research Institute, 01-138 Warsaw, Poland, e.kopec@igichp.edu.pl (E.A-K), a.zabost@igichp.edu.pl (A.Z)

[6] Department of Pharmaceutical Microbiology, Centre for Preclinical Research, Medical University of Warsaw, 02-097 Warszawa, Poland; jstefanska@wum.edu.pl (J.S.)

[7] Department of Biochemistry and Pharmacogenomics, Faculty of Pharmacy, Medical University of Warsaw, 02-097 Warsaw, Poland: mwrzosek@wum.edu.pl (M.W)

[*]Corresponding authors: Anna Bielenica; abielenica@wum.edu.pl (biological activity), Piotr Roszkowski; roszkowski@chem.uw.edu.pl (chemistry)

Below ^{1}H and ^{13}C NMR spectra of synthesized amides of MXF are presented.

The NMR analysis shown that studied derivatives of MXF and fatty acids existed in solution as a rotamers mixture. Therefore, in description of NMR spectra the major form was marked as a R_A and minor form was marked as R_B. In $CDCl_3$ the ratio of R_A:R_B is about 2:1 and in a case when the signals in NMR spectra were appropriate separated remarks were added to description. Interestingly, the propagation of rotamers depended on solvent used in NMR analysis and in case of DMSO-d_6 the ratio of R_A:R_B is about 1:1.

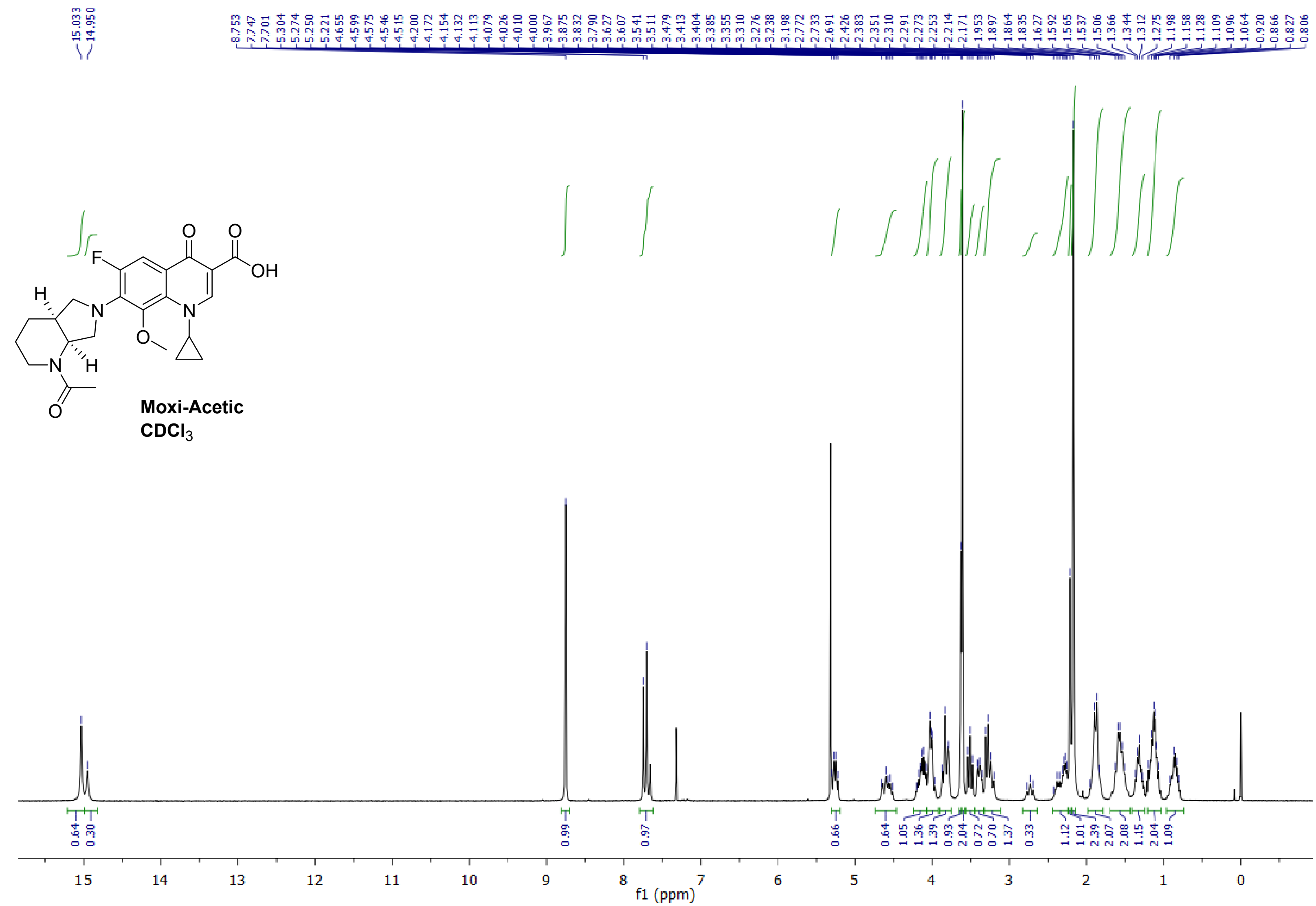

Moxi-Acetic
CDCl3
f1 (ppm)

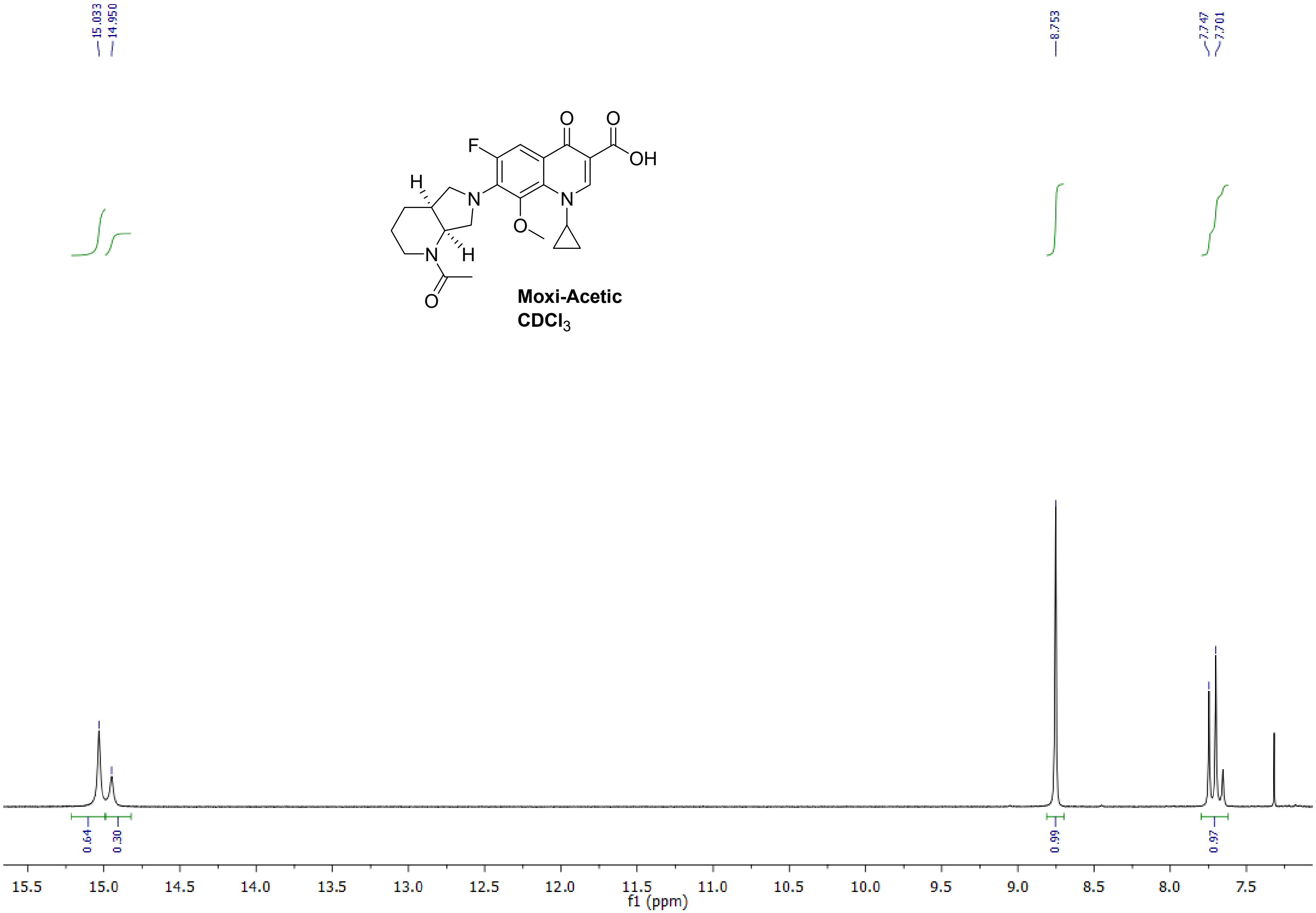
15.033
14.950
8.753
7.747
7.701
F
O
O
OH
N
N
H
H
N
O
O
Moxi-Acetic
CDCl3
0.64
0.30
0.99
0.97
15.5
15.0
14.5
14.0
13.5
13.0
12.5
12.0
11.5
11.0
10.5
10.0
9.5
9.0
8.5
8.0
7.5
f1 (ppm)

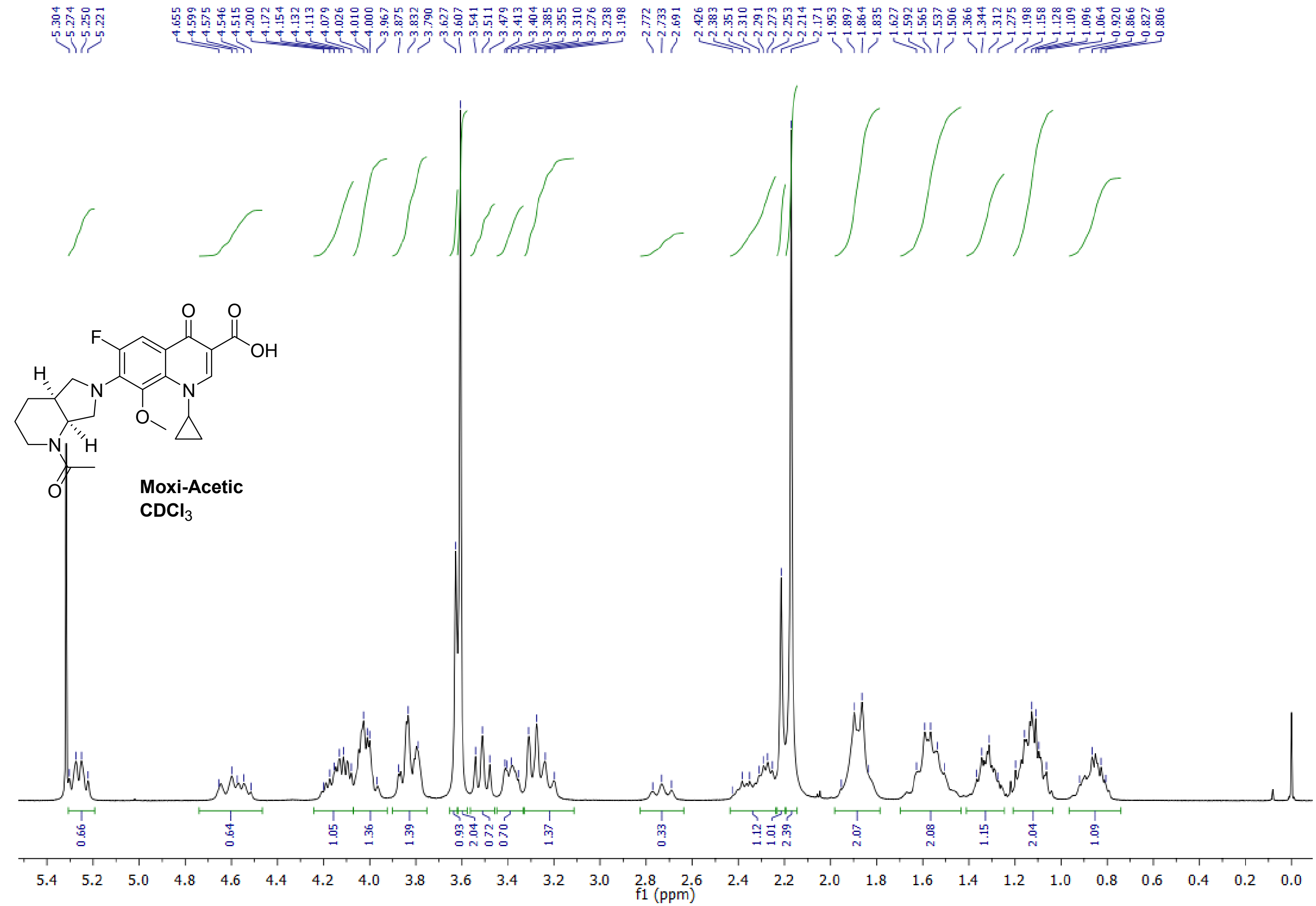
Moxi-Acetic
CDCl3
f1 (ppm)

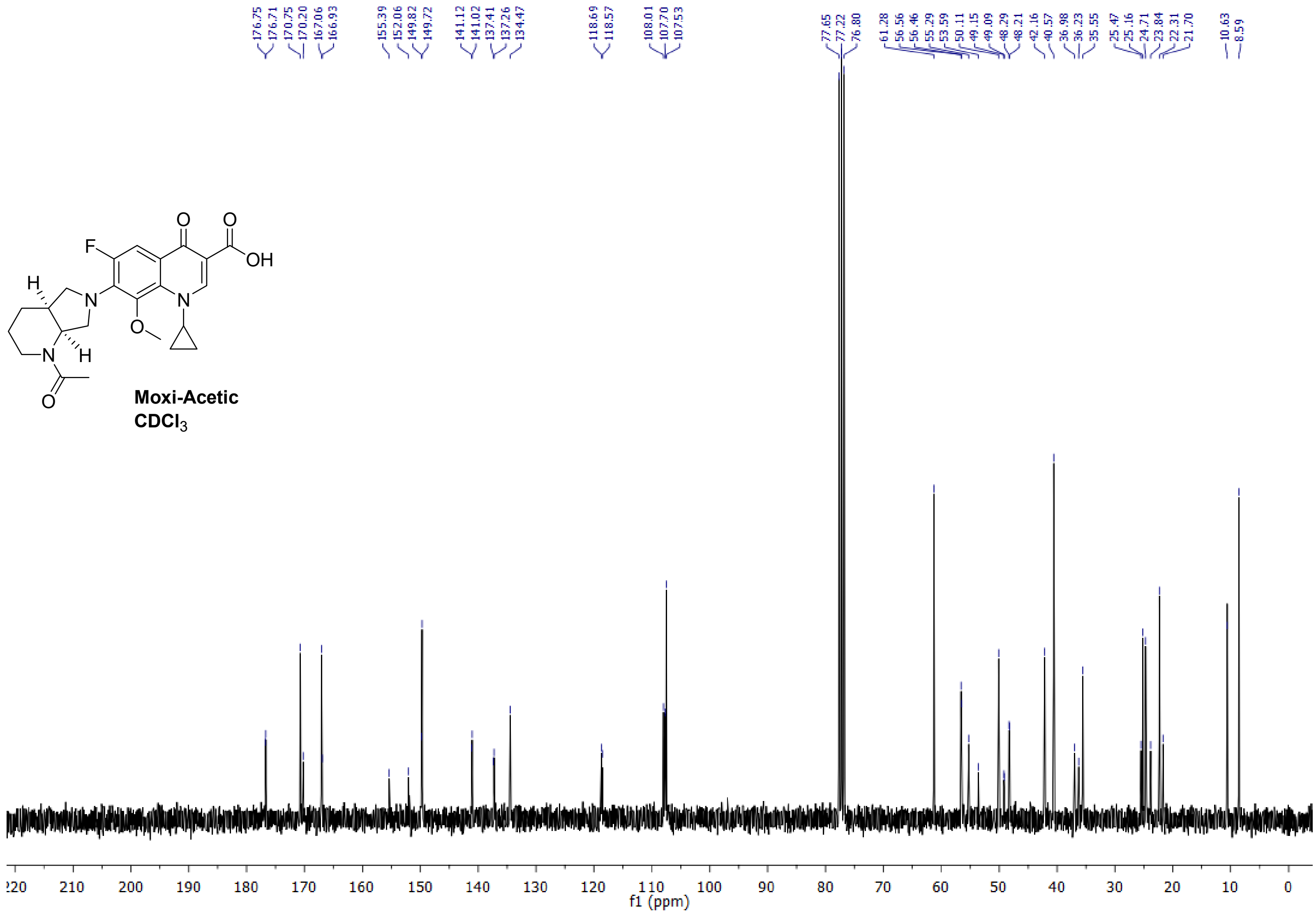
Moxi-Acetic
$CDCl_3$
f1 (ppm)

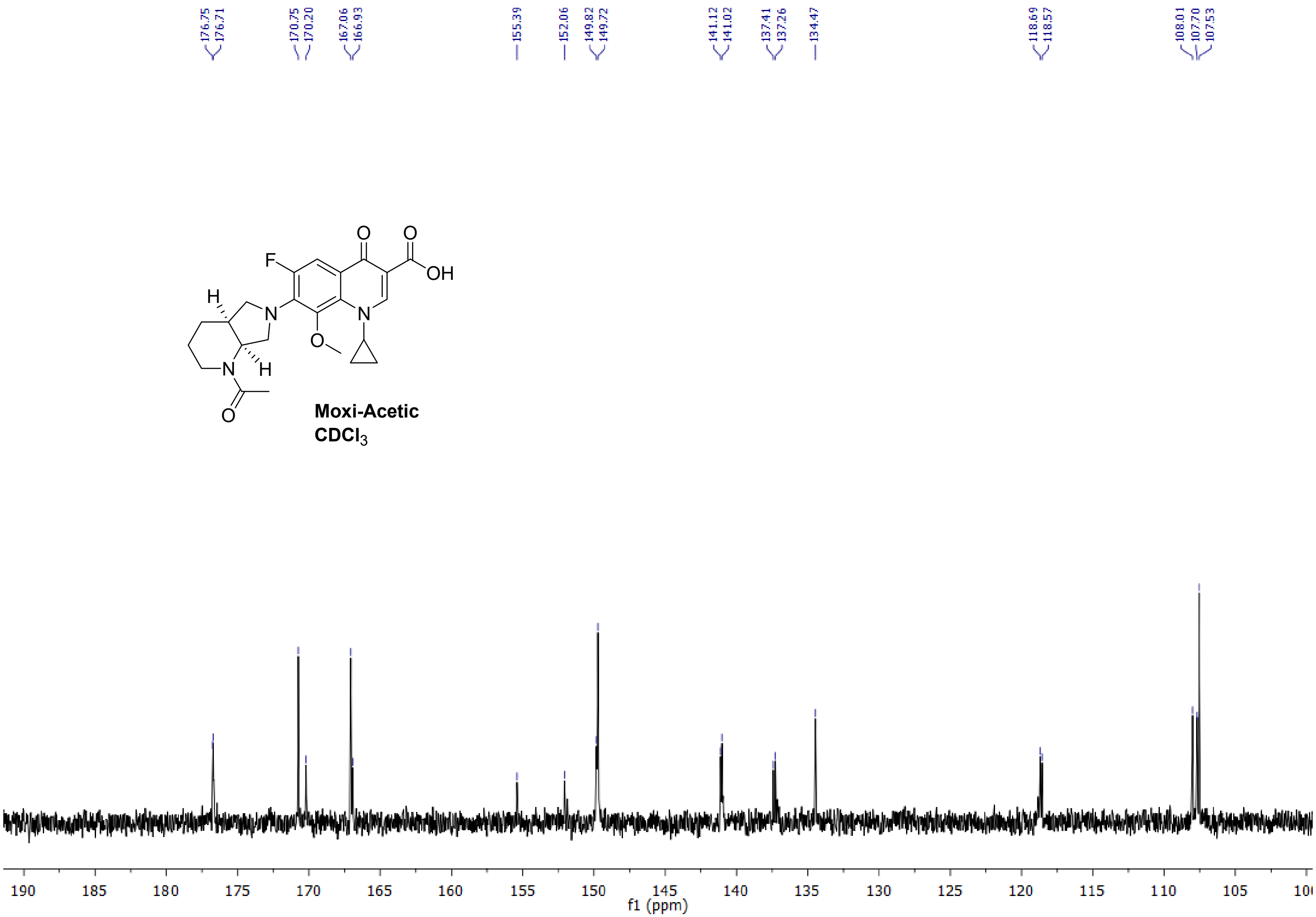
176.75
176.71
170.75
170.20
167.06
166.93
155.39
152.06
149.82
149.72
141.12
141.02
137.41
137.26
134.47
118.69
118.57
108.01
107.70
107.53
F
O
O
OH
H
N
N
O
H
N
O
Moxi-Acetic
$CDCl_3$
190
185
180
175
170
165
160
155
150
145
140
135
130
125
120
115
110
105
f1 (ppm)

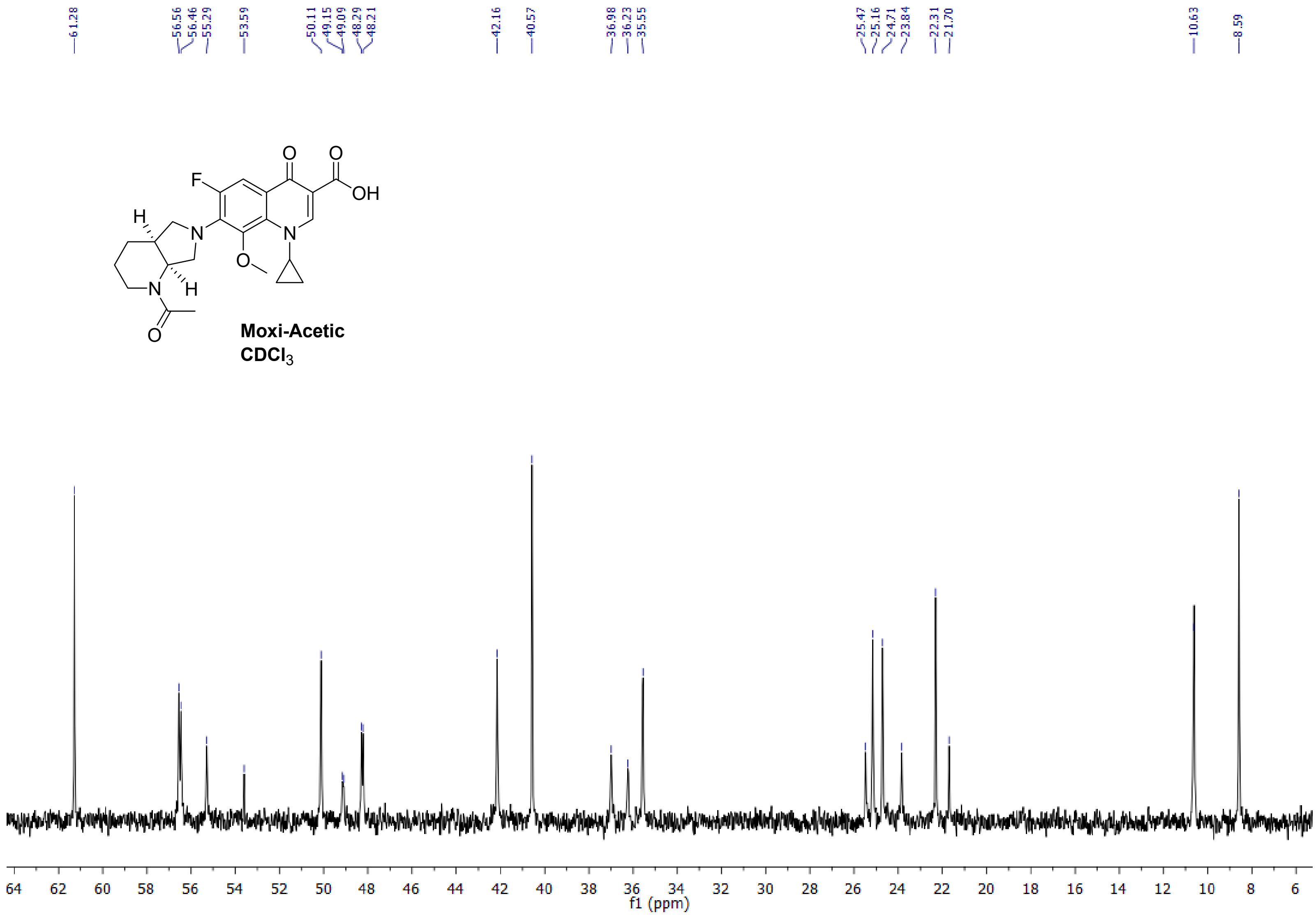

61.28
56.56
56.46
55.29
53.59
50.11
49.15
49.09
48.29
48.21
42.16
40.57
36.98
36.23
35.55
25.47
25.16
24.71
23.84
22.31
21.70
10.63
8.59
Moxi-Acetic
CDCl3
64 62 60 58 56 54 52 50 48 46 44 42 40 38 36 34 32 30 28 26 24 22 20 18 16 14 12 10 8 6
f1 (ppm)

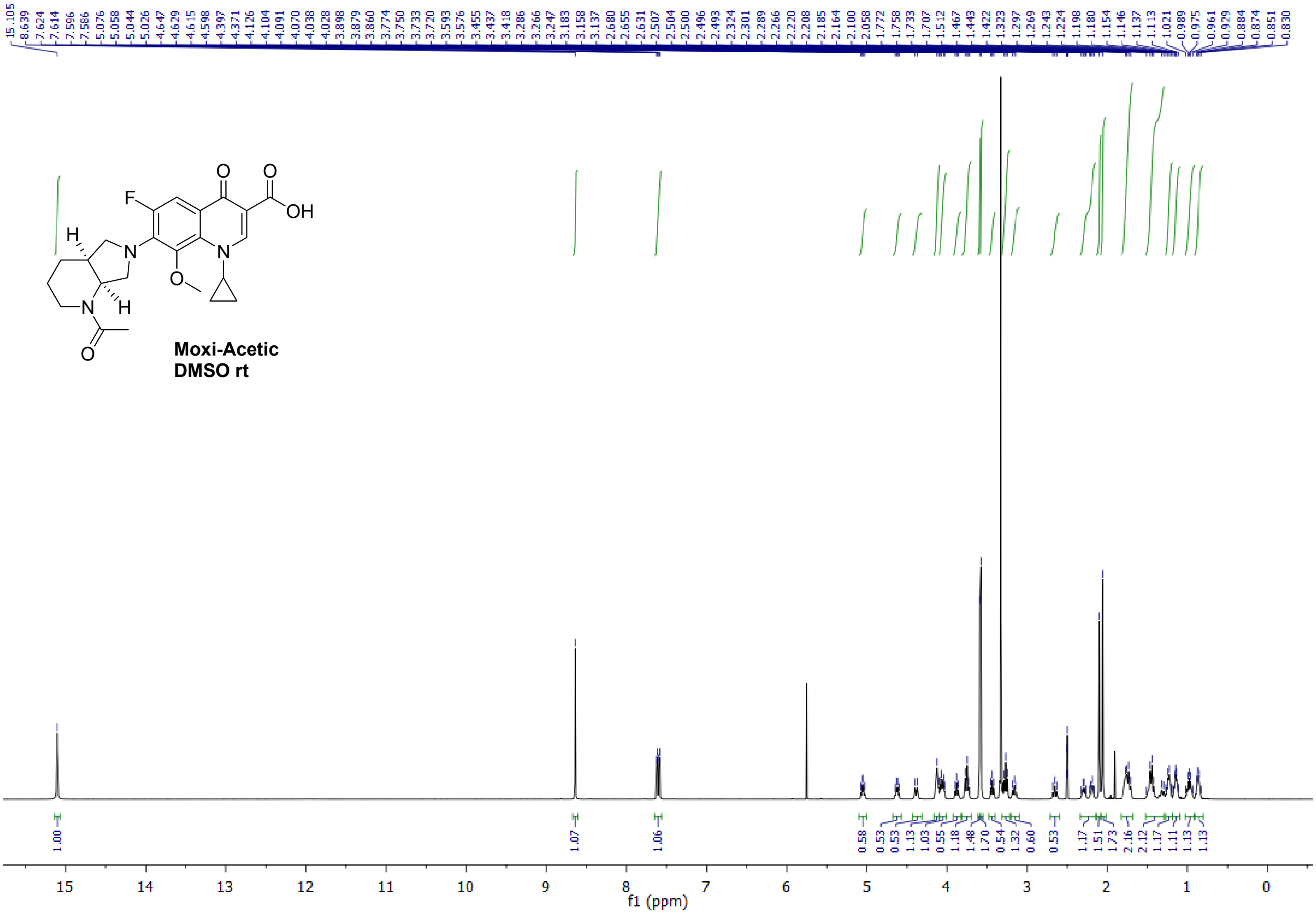
Moxi-Acetic
DMSO rt
f1 (ppm)

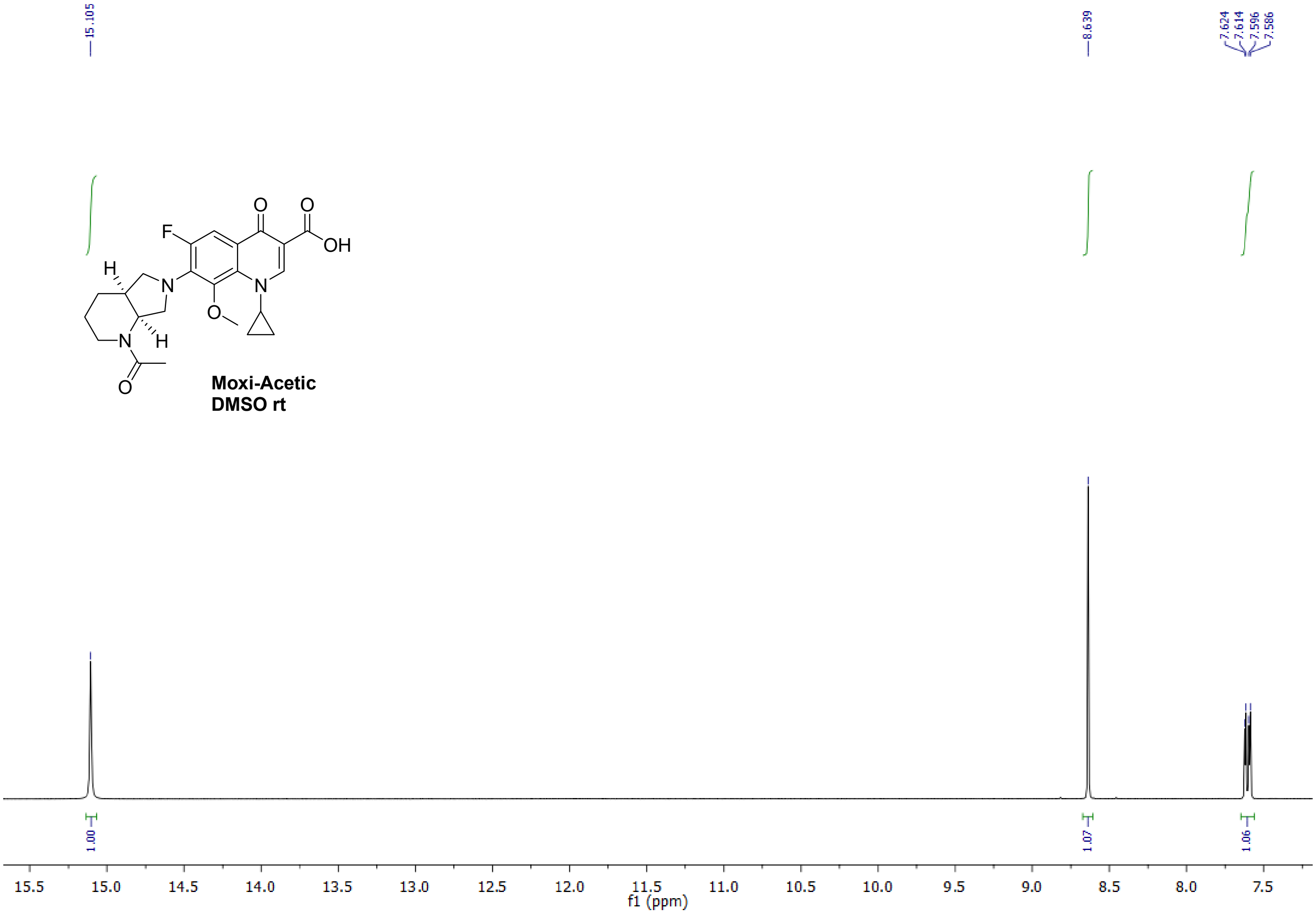
15.105
8.639
7.624
7.614
7.596
7.586
Moxi-Acetic
DMSO rt
1.00
1.07
1.06
15.5
15.0
14.5
14.0
13.5
13.0
12.5
12.0
11.5
11.0
10.5
10.0
9.5
9.0
8.5
8.0
7.5
f1 (ppm)

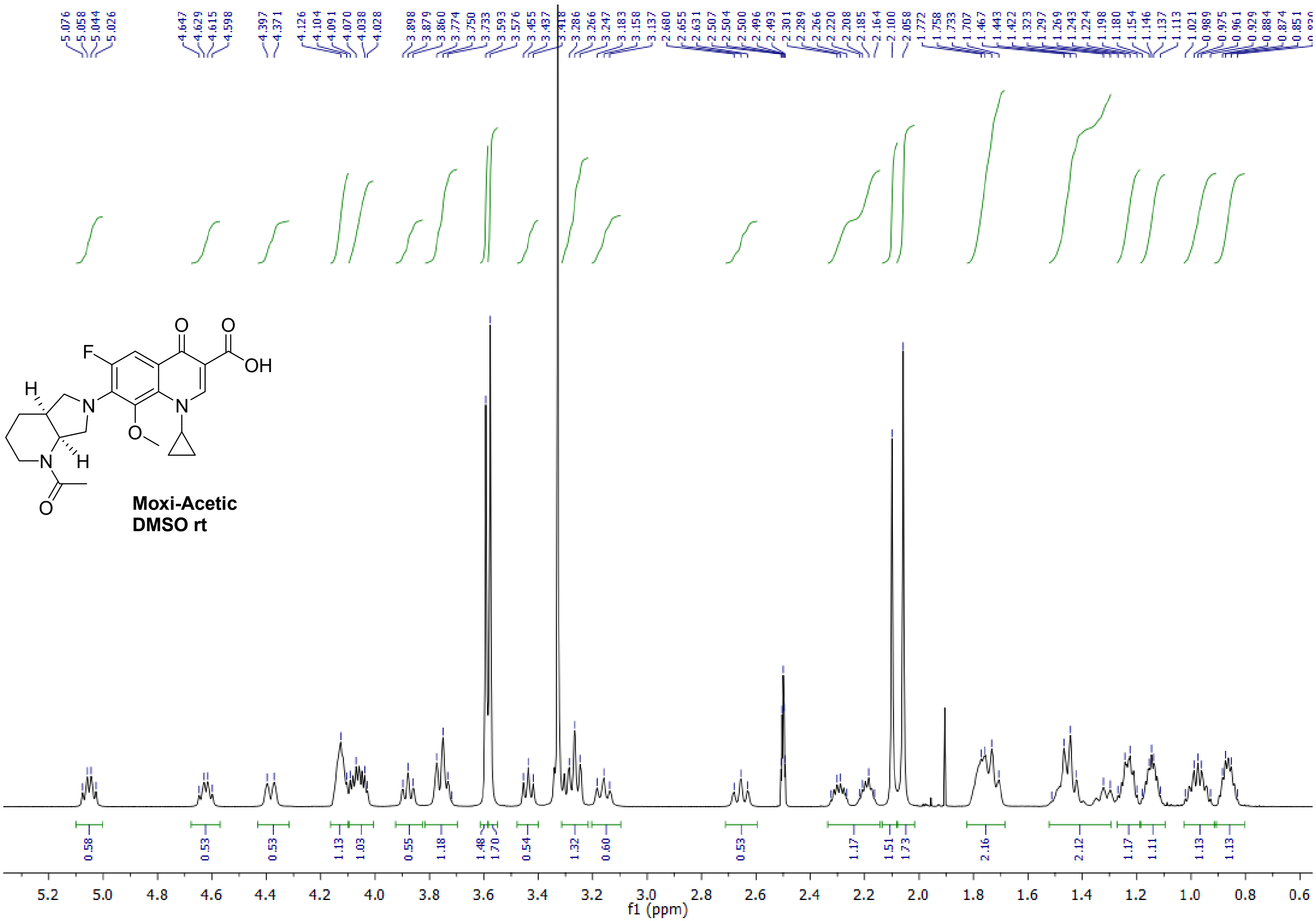
Moxi-Acetic
DMSO rt
f1 (ppm)

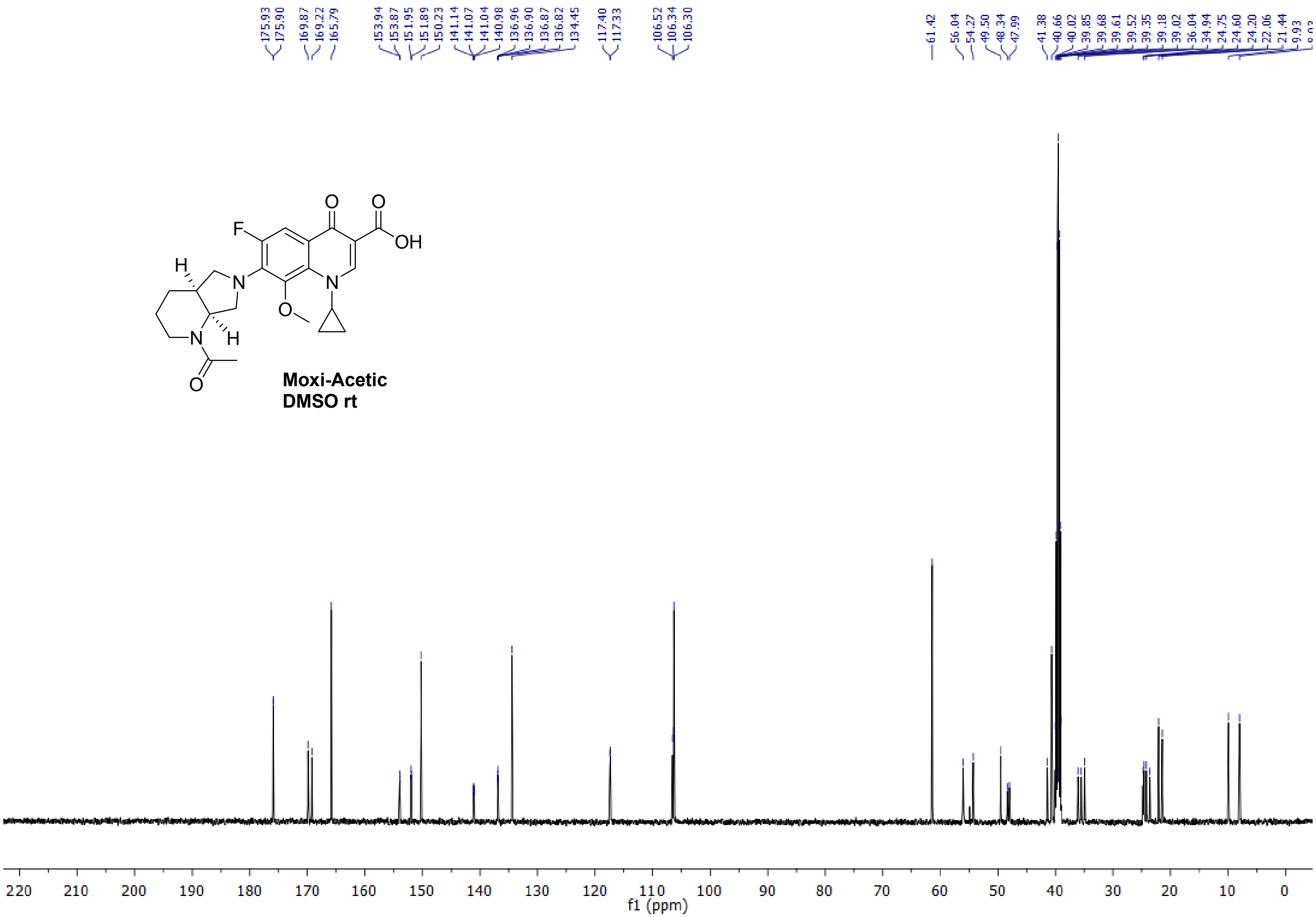
175.93
175.90
169.87
169.22
165.79
153.94
153.87
151.95
151.89
150.23
141.14
141.07
141.04
140.98
136.96
136.90
136.87
136.82
134.45
117.40
117.33
106.52
106.34
106.30
61.42
56.04
54.27
49.50
48.34
47.99
41.38
40.66
40.02
39.85
39.68
39.61
39.52
39.35
39.18
39.02
36.04
34.94
24.75
24.60
24.20
22.06
21.44
9.93
F
O
O
OH
N
H
N
O
H
N
O
Moxi-Acetic
DMSO rt
220
210
200
190
180
170
160
150
140
130
120
110
100
90
80
70
60
50
40
30
20
10
0
f1 (ppm)

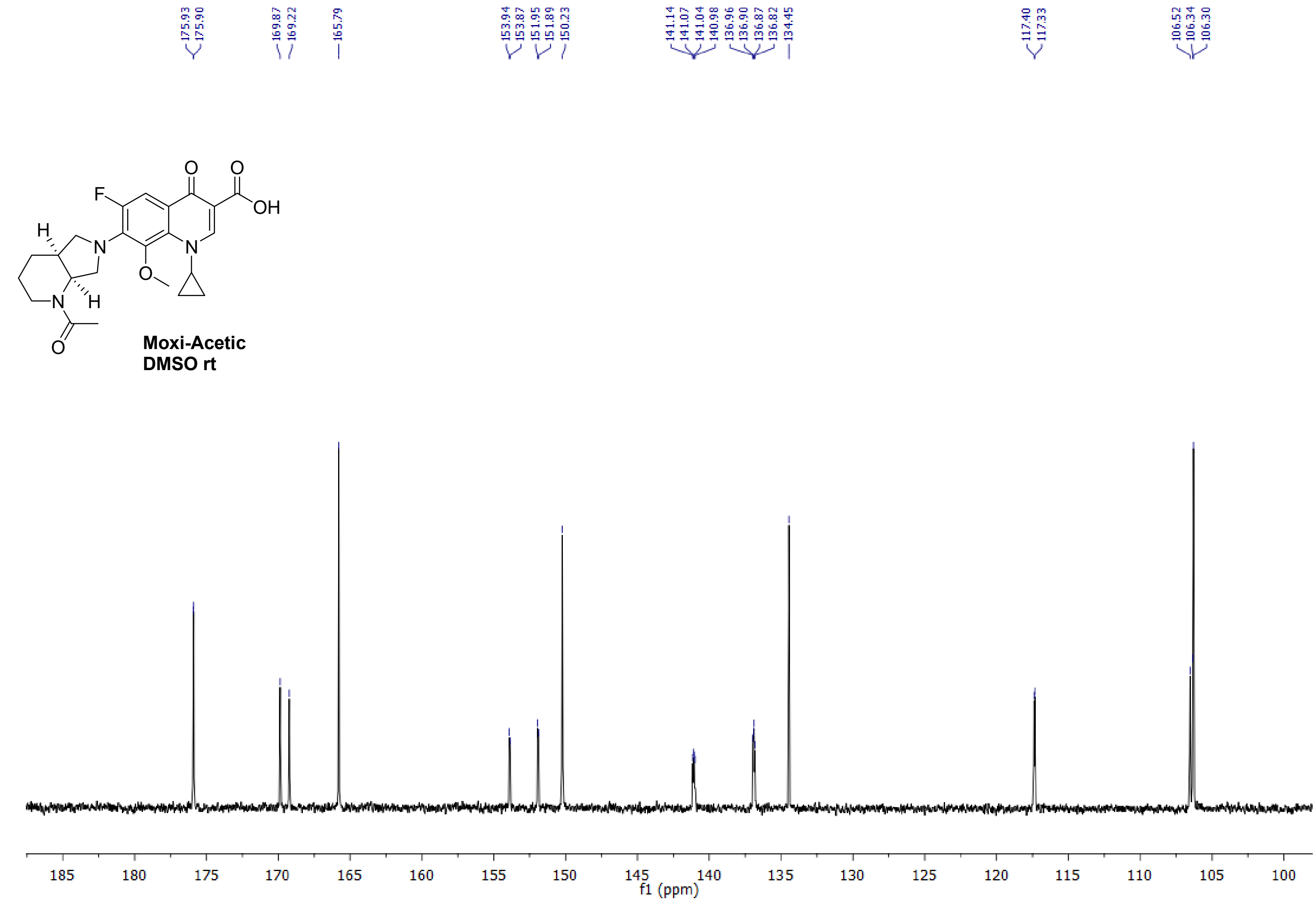

Moxi-Acetic
DMSO rt

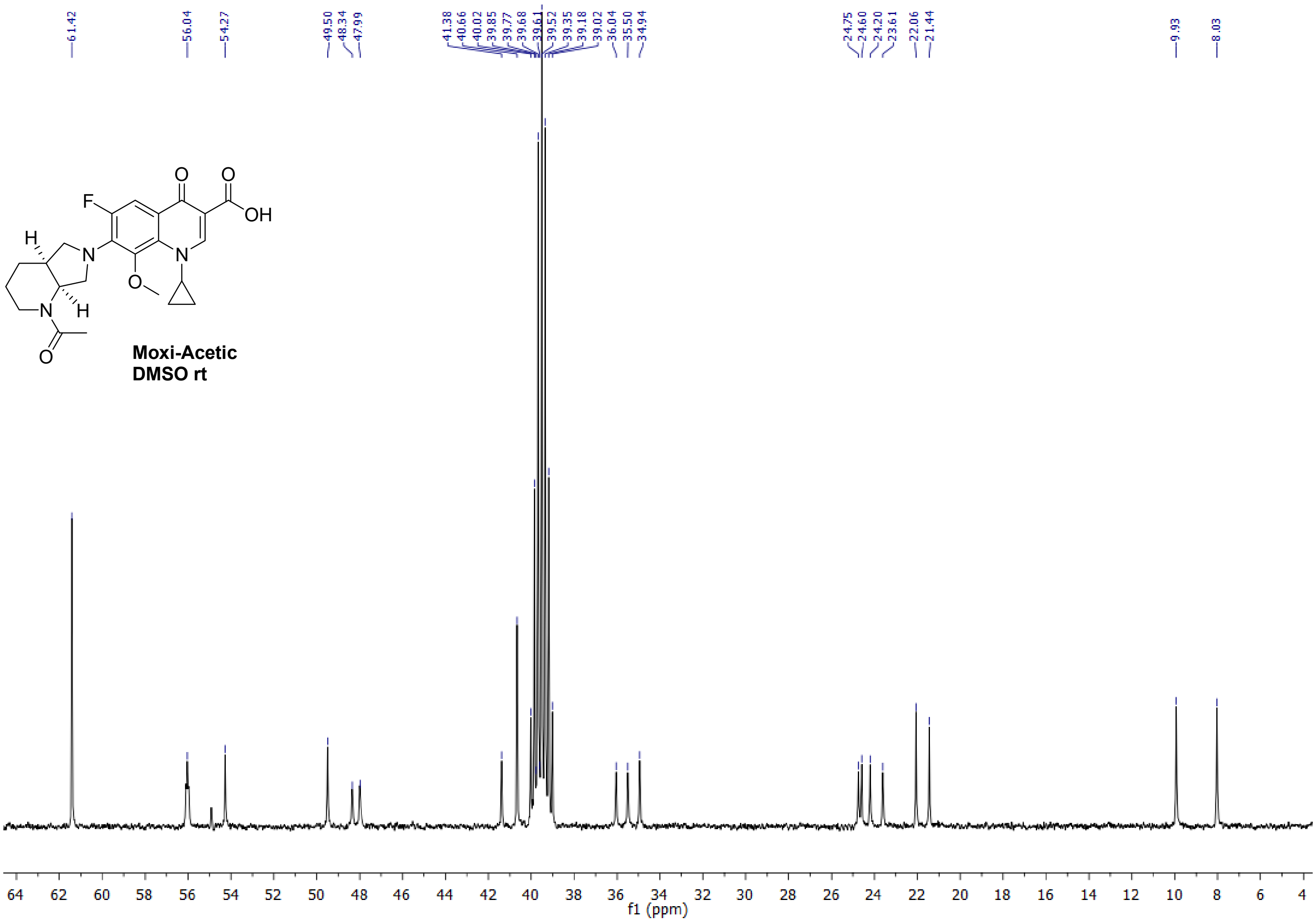

61.42
56.04
54.27
49.50
48.34
47.99
41.38
40.66
40.02
39.85
39.77
39.68
39.61
39.52
39.35
39.18
39.02
36.04
35.50
34.94
24.75
24.60
24.20
23.61
22.06
21.44
9.93
8.03
Moxi-Acetic
DMSO rt
f1 (ppm)

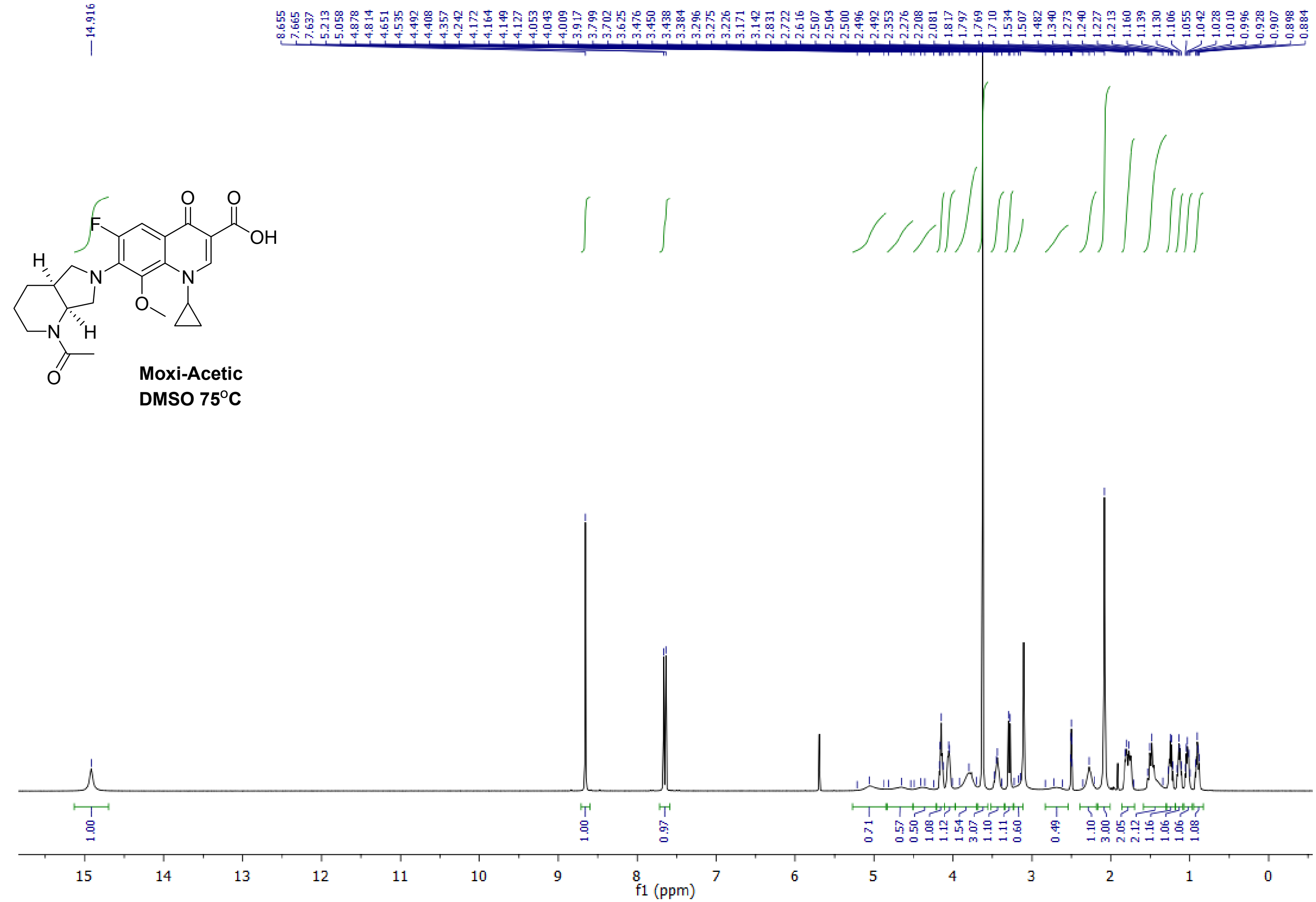

Moxi-Acetic
DMSO 75°C

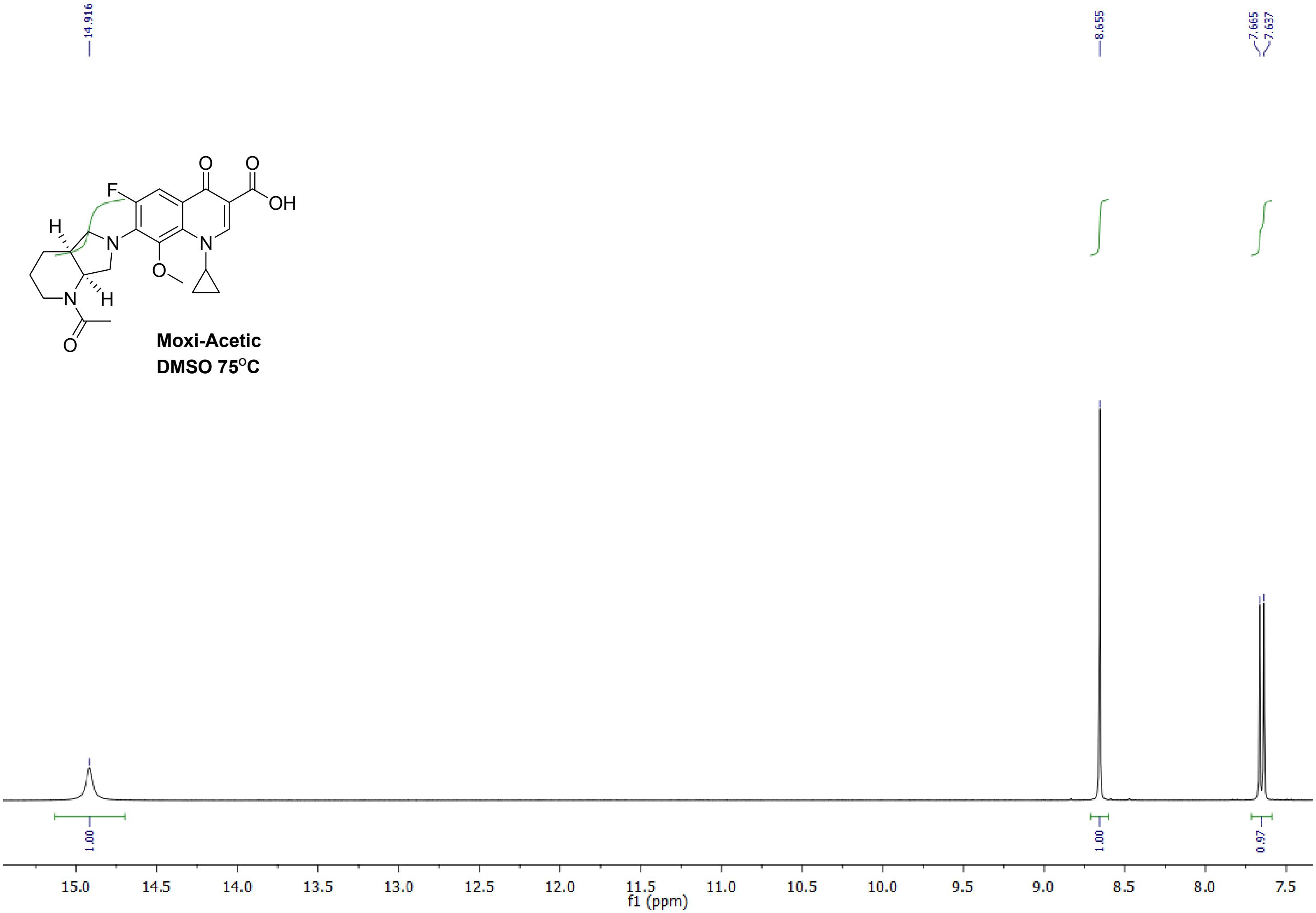
14.916
8.655
7.665
7.637
Moxi-Acetic
DMSO 75°C
1.00
1.00
0.97
15.0
14.5
14.0
13.5
13.0
12.5
12.0
11.5
11.0
10.5
10.0
9.5
9.0
8.5
8.0
7.5
f1 (ppm)

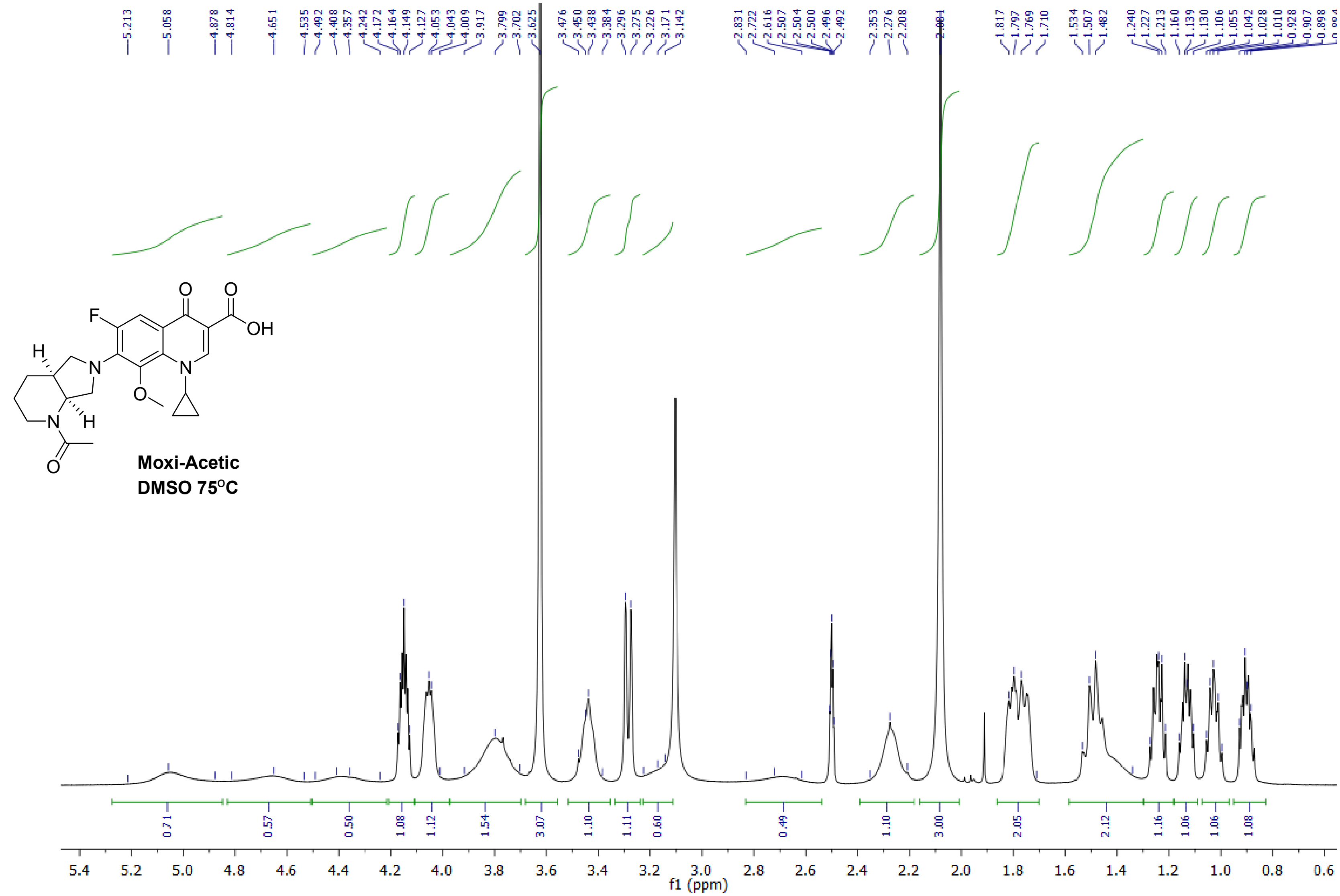

Moxi-Acetic
DMSO 75°C

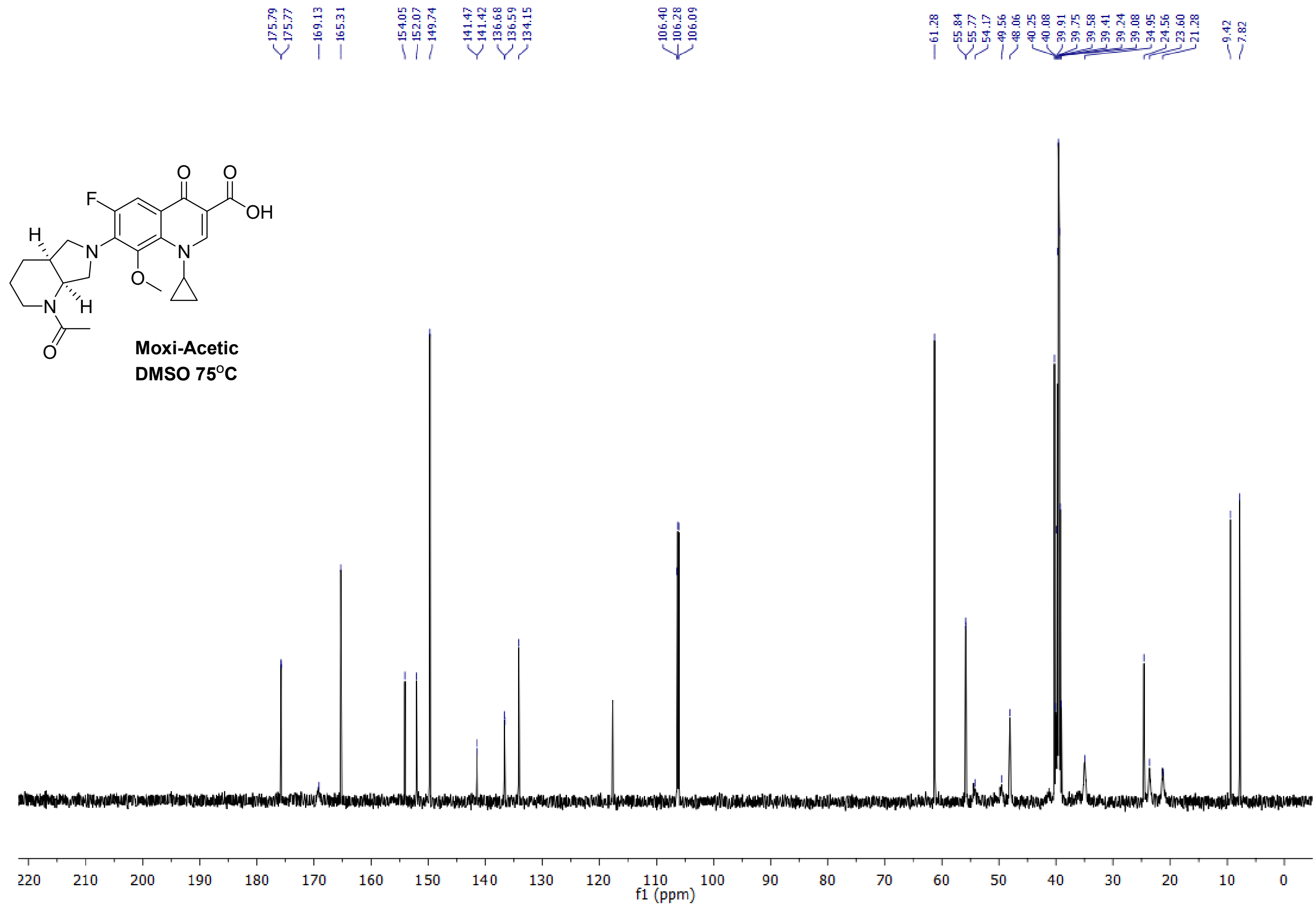
175.79
175.77
169.13
165.31
154.05
152.07
149.74
141.47
141.42
136.68
136.59
134.15
106.40
106.28
106.09
61.28
55.84
55.77
54.17
49.56
48.06
40.25
40.08
39.91
39.75
39.58
39.41
39.24
39.08
34.95
24.56
23.60
21.28
9.42
7.82
F
O
OH
N
H
Moxi-Acetic
DMSO 75°C
f1 (ppm)

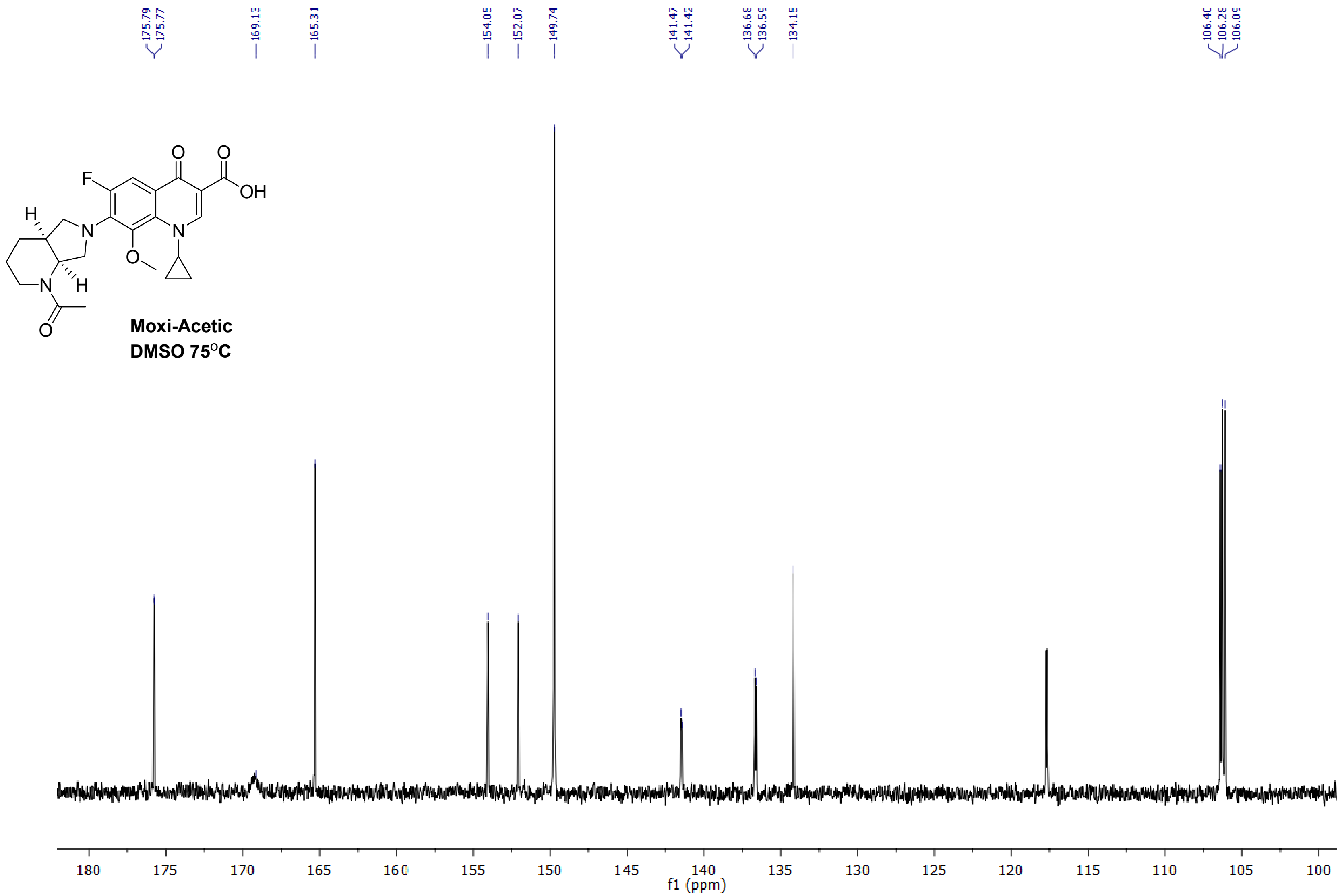

175.79
175.77
169.13
165.31
154.05
152.07
149.74
141.47
141.42
136.68
136.59
134.15
106.40
106.28
106.09
Moxi-Acetic
DMSO 75°C
180
175
170
165
160
155
150
145
140
135
130
125
120
115
110
105
100
f1 (ppm)

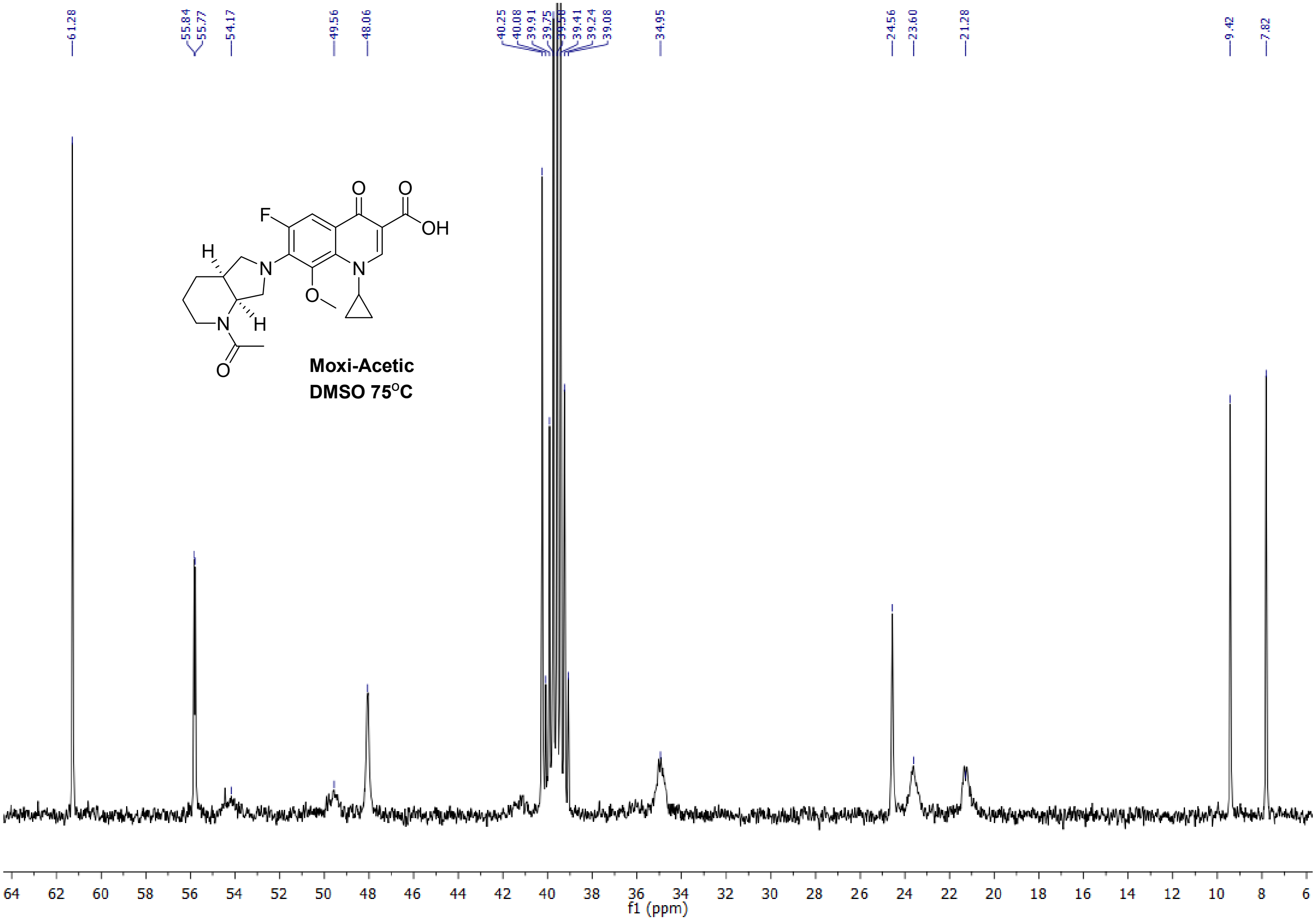

Moxi-Acetic
DMSO 75°C
f1 (ppm)

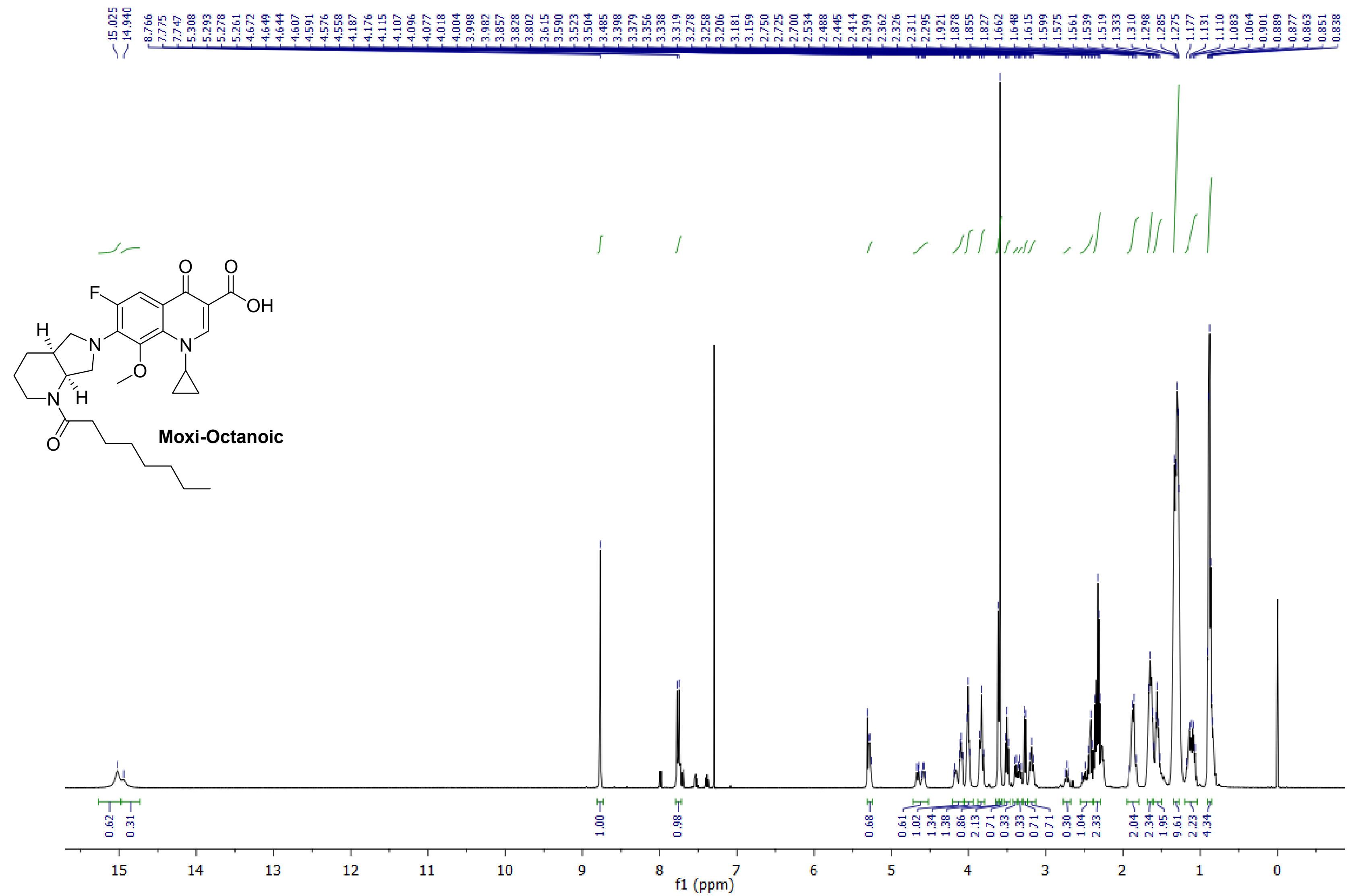
Moxi-Octanoic
f1 (ppm)

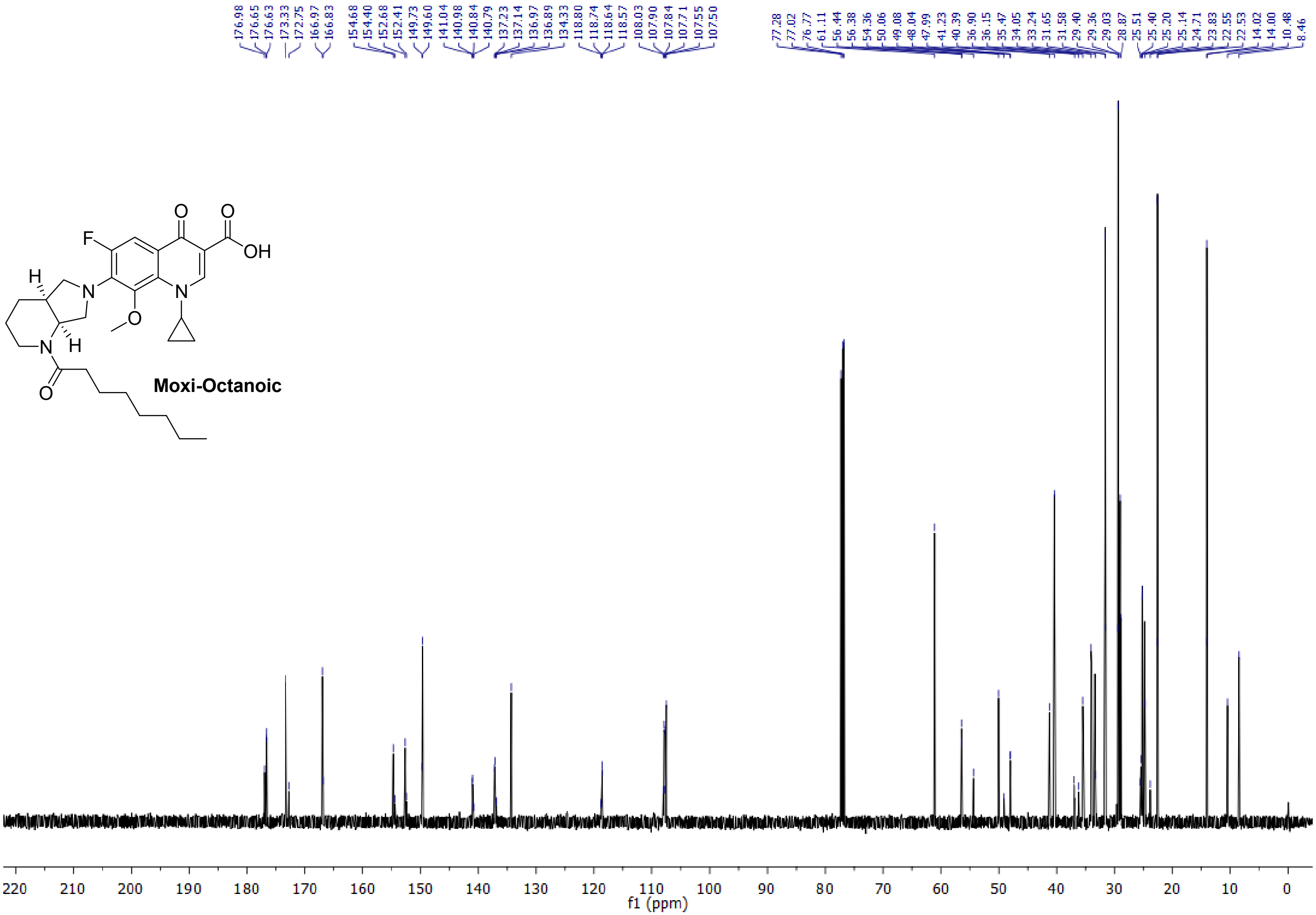
176.98
176.65
176.63
173.33
172.75
166.97
166.83
154.68
154.40
152.68
152.41
149.73
149.60
141.04
140.98
140.84
140.79
137.23
137.14
136.97
136.89
134.33
118.80
118.74
118.64
118.57
108.03
107.90
107.84
107.71
107.55
107.50
77.28
77.02
76.77
61.11
56.44
56.38
54.36
50.06
49.08
48.04
47.99
41.23
40.39
36.90
36.15
35.47
34.05
33.24
31.65
31.58
29.40
29.36
29.03
28.87
25.51
25.40
25.20
25.14
24.71
23.83
22.55
22.53
14.02
14.00
10.48
8.46
F
O
O
OH
H
N
N
O
N
H
O
Moxi-Octanoic
220
210
200
190
180
170
160
150
140
130
120
110
100
90
80
70
60
50
40
30
20
10
0
f1 (ppm)

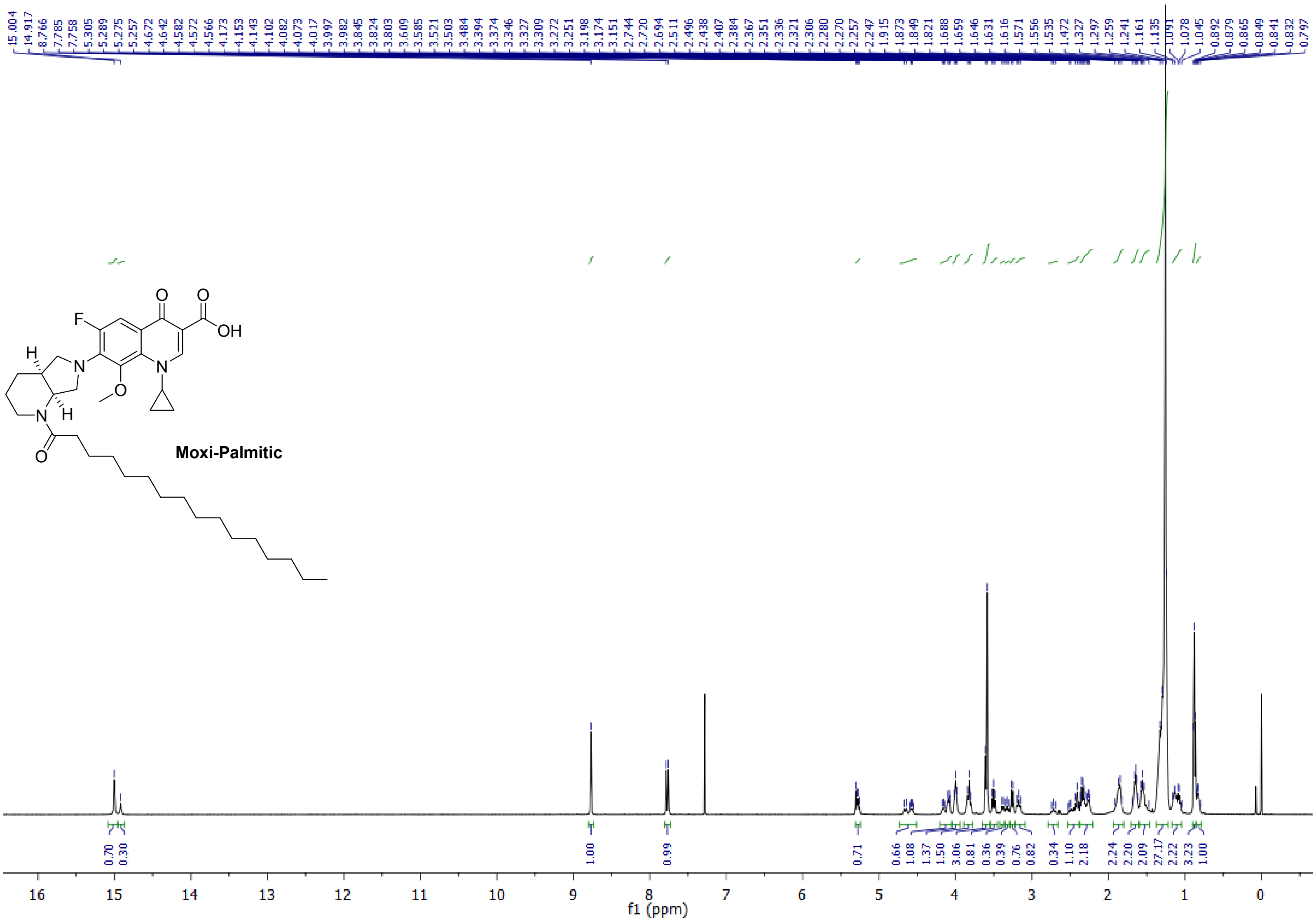

Moxi-Palmitic
f1 (ppm)

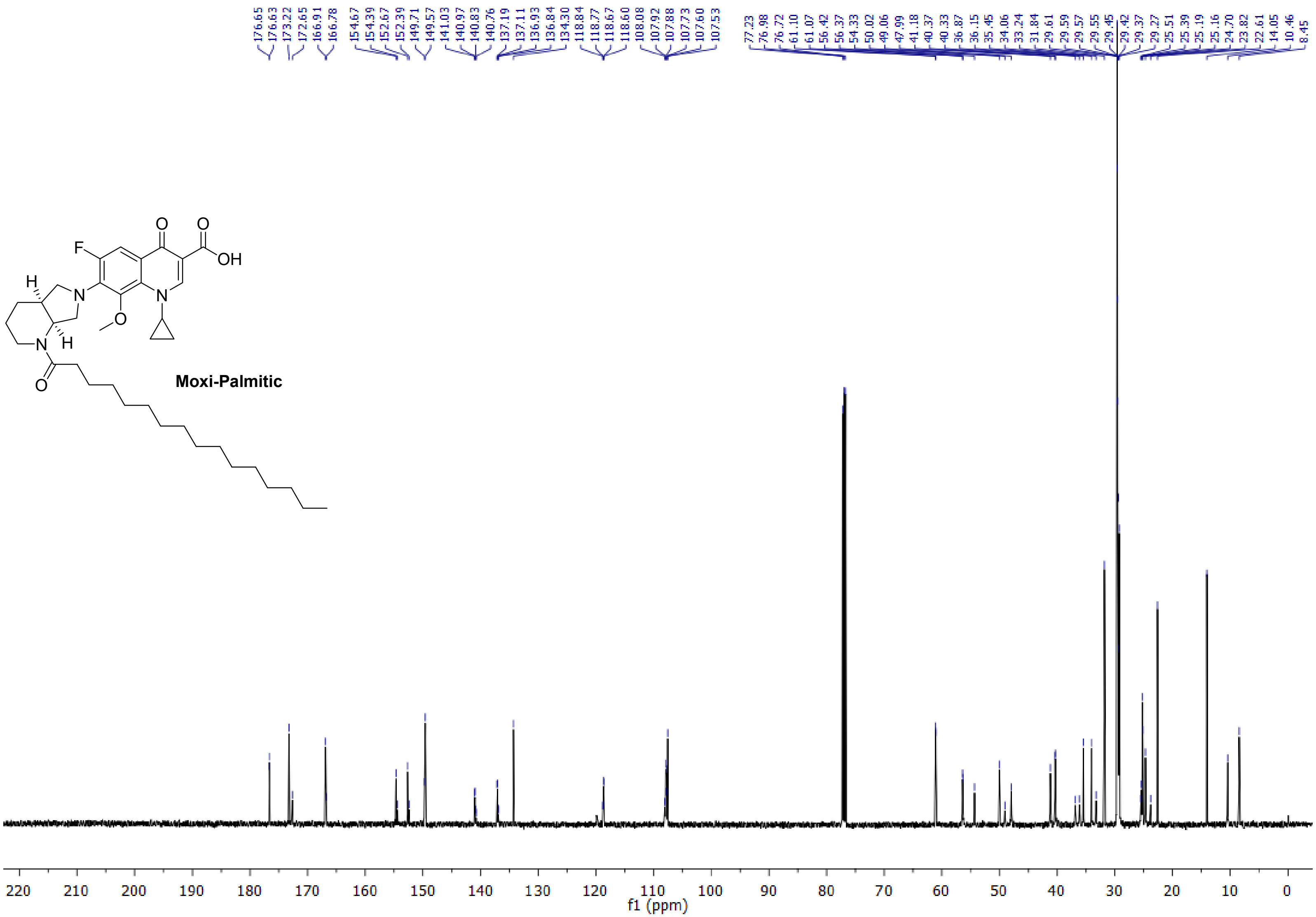
176.65
176.63
173.22
172.65
166.91
166.78
154.67
154.39
152.67
152.39
149.71
149.57
141.03
140.97
140.83
140.76
137.19
137.11
136.93
136.84
134.30
118.84
118.77
118.67
118.60
108.08
107.92
107.88
107.73
107.60
107.53
77.23
76.98
76.72
61.10
61.07
56.42
56.37
54.33
50.02
49.06
47.99
41.18
40.37
40.33
36.87
36.15
35.45
34.06
33.24
31.84
29.61
29.59
29.57
29.55
29.45
29.42
29.37
29.27
25.51
25.39
25.19
25.16
24.70
23.82
22.61
14.05
10.46
8.45
F
O
O
OH
H
N
N
O
H
N
O
Moxi-Palmitic
220 210 200 190 180 170 160 150 140 130 120 110 100 90 80 70 60 50 40 30 20 10 0
f1 (ppm)

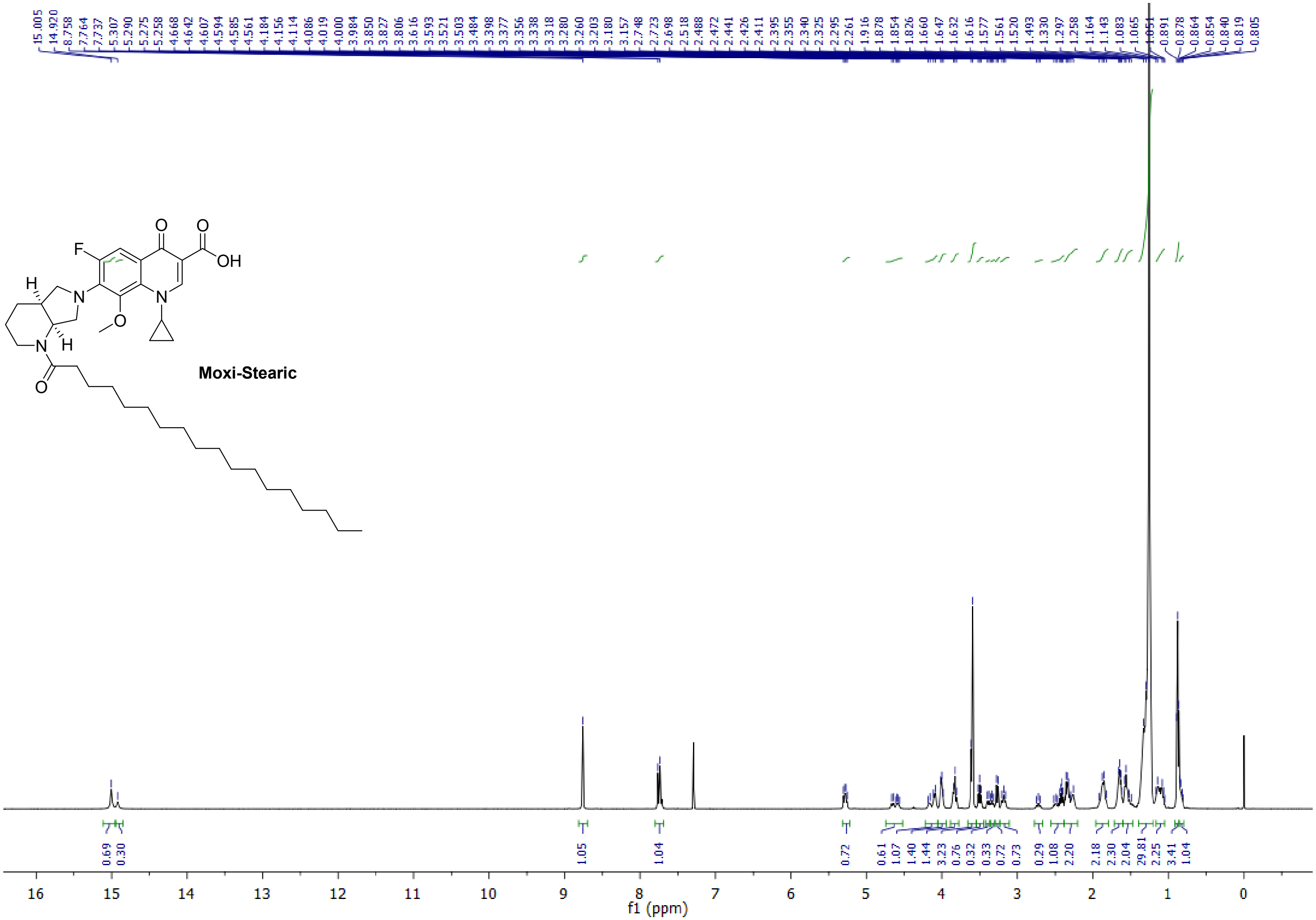
Moxi-Stearic
f1 (ppm)

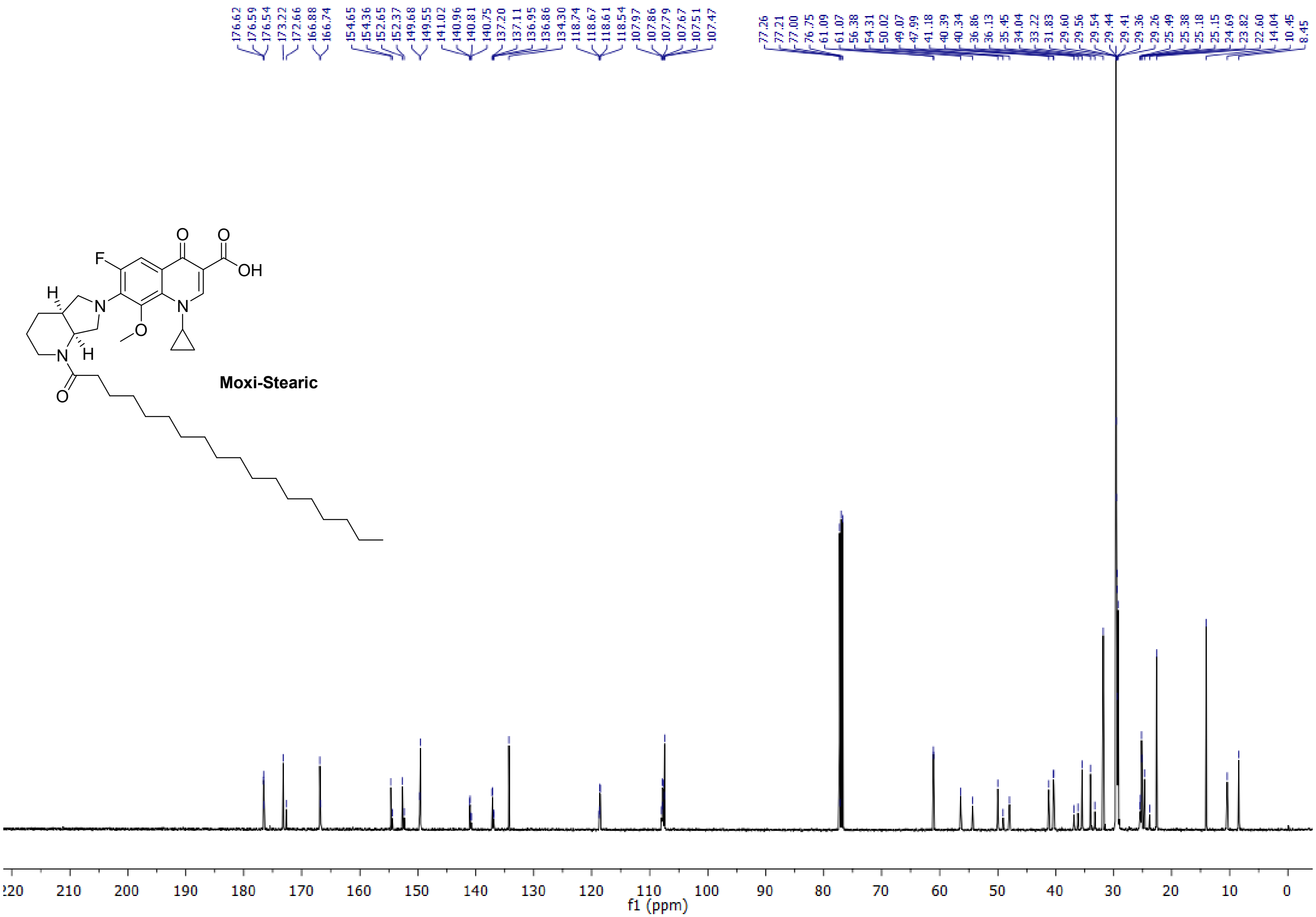
176.62
176.59
176.54
173.22
172.66
166.88
166.74
154.65
154.36
152.65
152.37
149.68
149.55
141.02
140.96
140.81
140.75
137.20
137.11
136.95
136.86
134.30
118.74
118.67
118.61
118.54
107.97
107.86
107.79
107.67
107.51
107.47
77.26
77.21
77.00
76.75
61.09
61.07
56.38
54.31
50.02
49.07
47.99
41.18
40.39
40.34
36.86
36.13
35.45
34.04
33.22
31.83
29.60
29.56
29.54
29.44
29.41
29.36
29.26
25.49
25.38
25.18
25.15
24.69
23.82
22.60
14.04
10.45
8.45
F
O
O
OH
H
N
N
O
H
N
O
Moxi-Stearic
220 210 200 190 180 170 160 150 140 130 120 110 100 90 80 70 60 50 40 30 20 10 0
f1 (ppm)

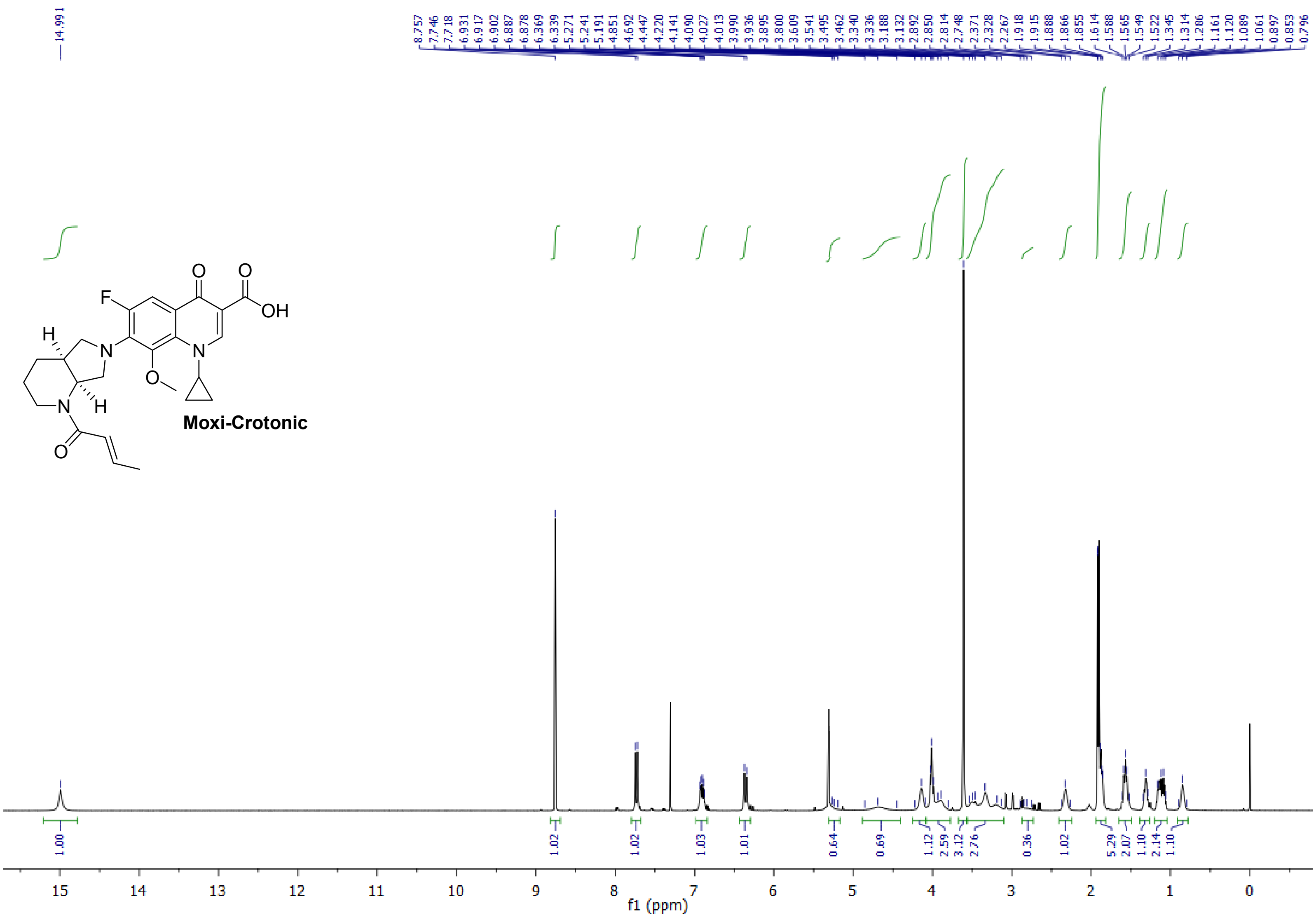
Moxi-Crotonic
f1 (ppm)

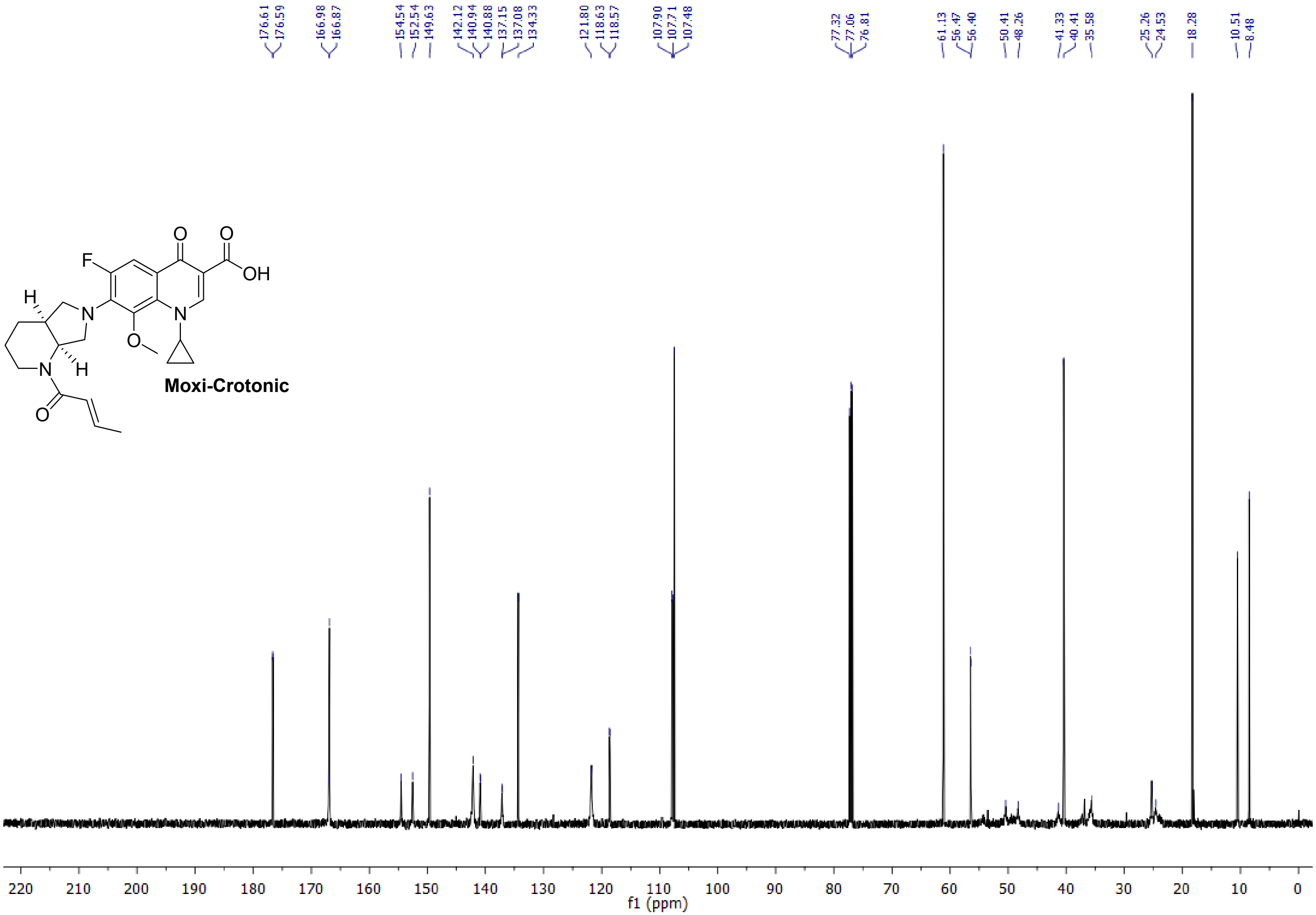
176.61
176.59
166.98
166.87
154.54
152.54
149.63
142.12
140.94
140.88
137.15
137.08
134.33
121.80
118.63
118.57
107.90
107.71
107.48
77.32
77.06
76.81
61.13
56.47
56.40
50.41
48.26
41.33
40.41
35.58
25.26
24.53
18.28
10.51
8.48
F
O
O
OH
H
N
N
O
N
H
O
Moxi-Crotonic
220
210
200
190
180
170
160
150
140
130
120
110
100
90
80
70
60
50
40
30
20
10
0
f1 (ppm)

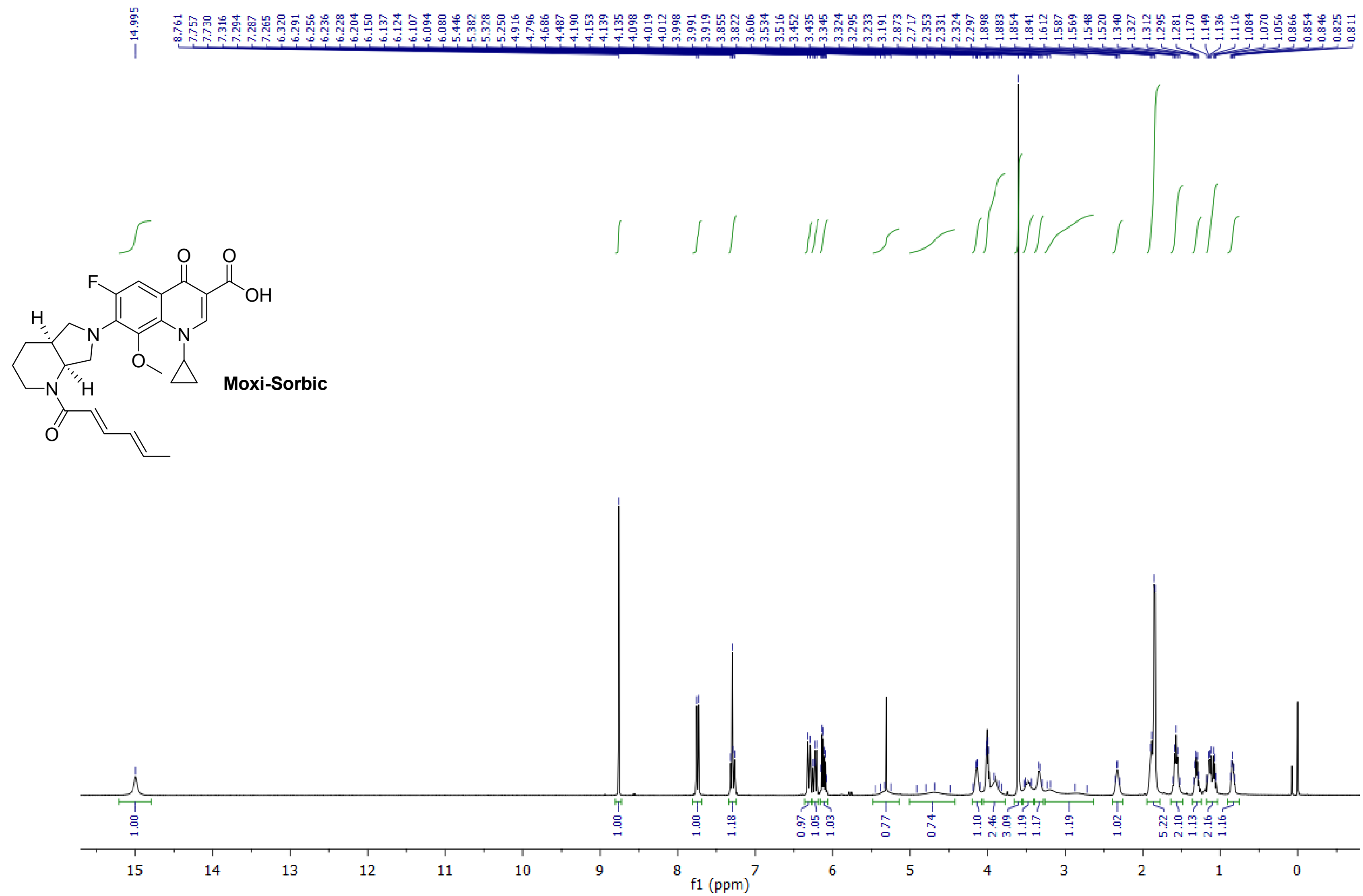

Moxi-Sorbic
f1 (ppm)

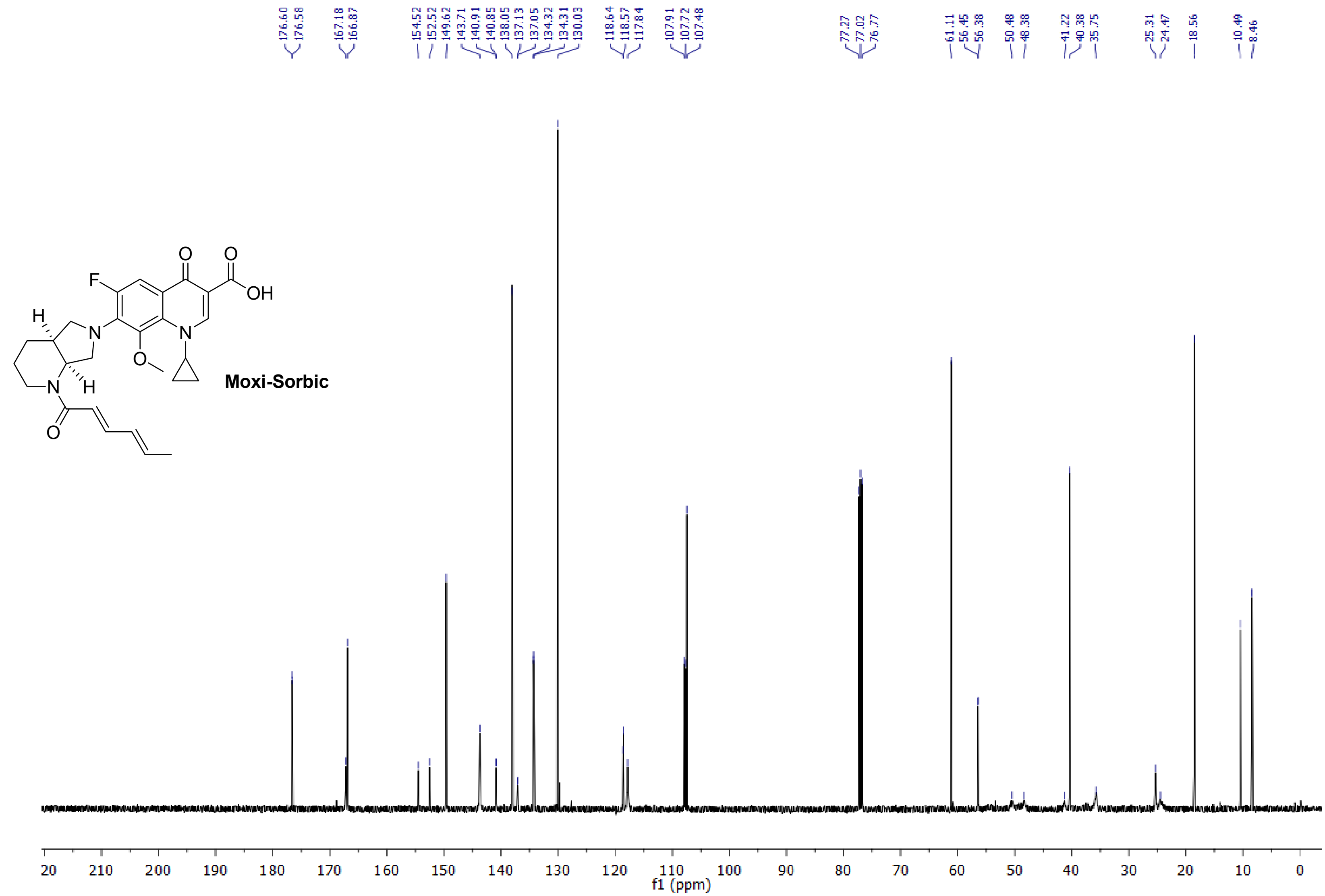
Moxi-Sorbic
176.60
176.58
167.18
166.87
154.52
152.52
149.62
143.71
140.91
140.85
138.05
137.13
137.05
134.32
134.31
130.03
118.64
118.57
117.84
107.91
107.72
107.48
77.27
77.02
76.77
61.11
56.45
56.38
50.48
48.38
41.22
40.38
35.75
25.31
24.47
18.56
10.49
8.46
f1 (ppm)

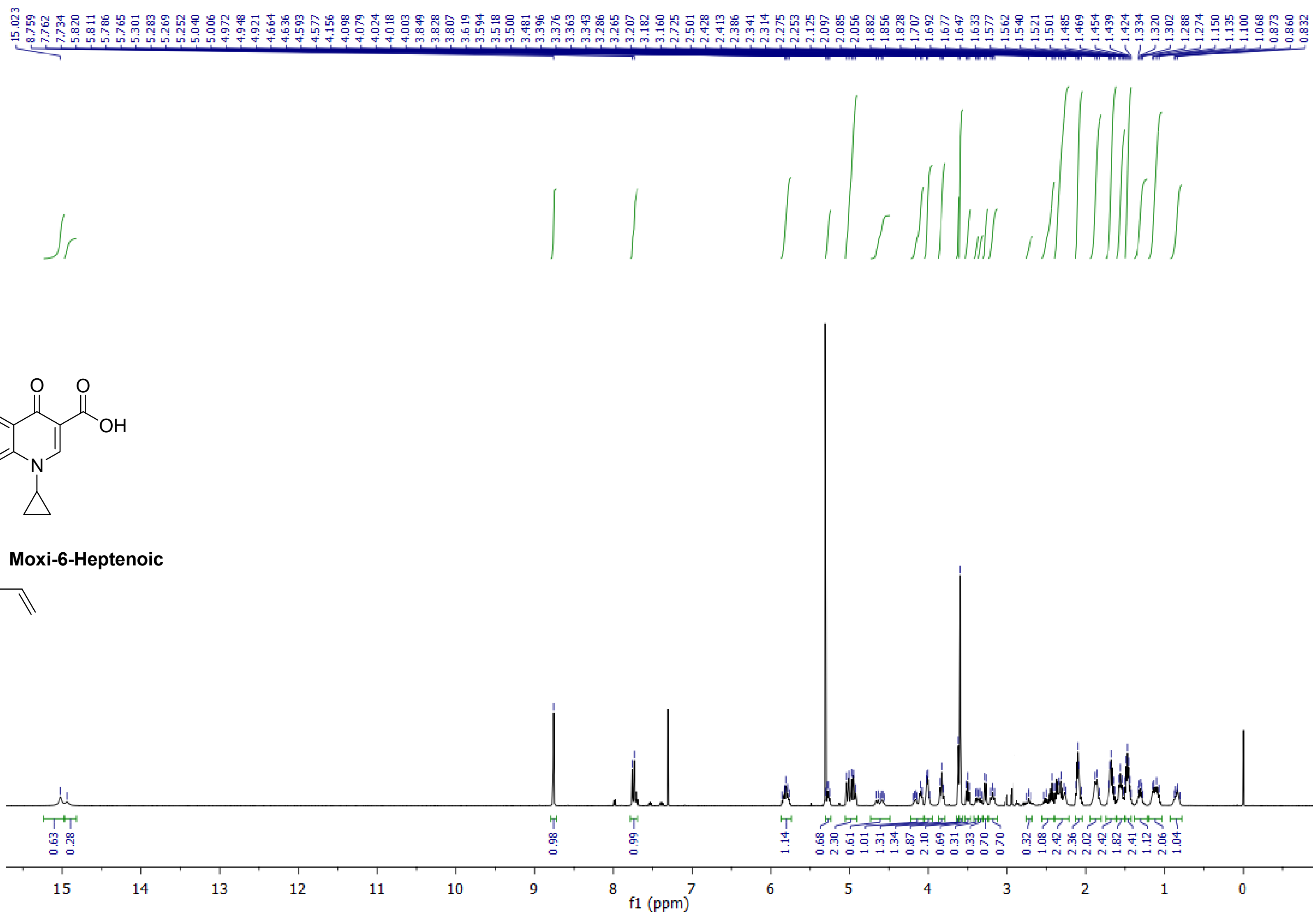

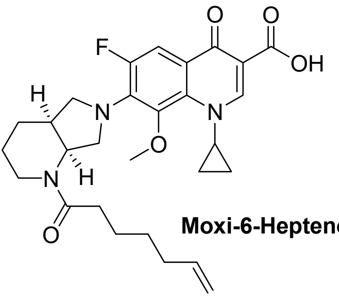

Moxi-6-Heptenoic

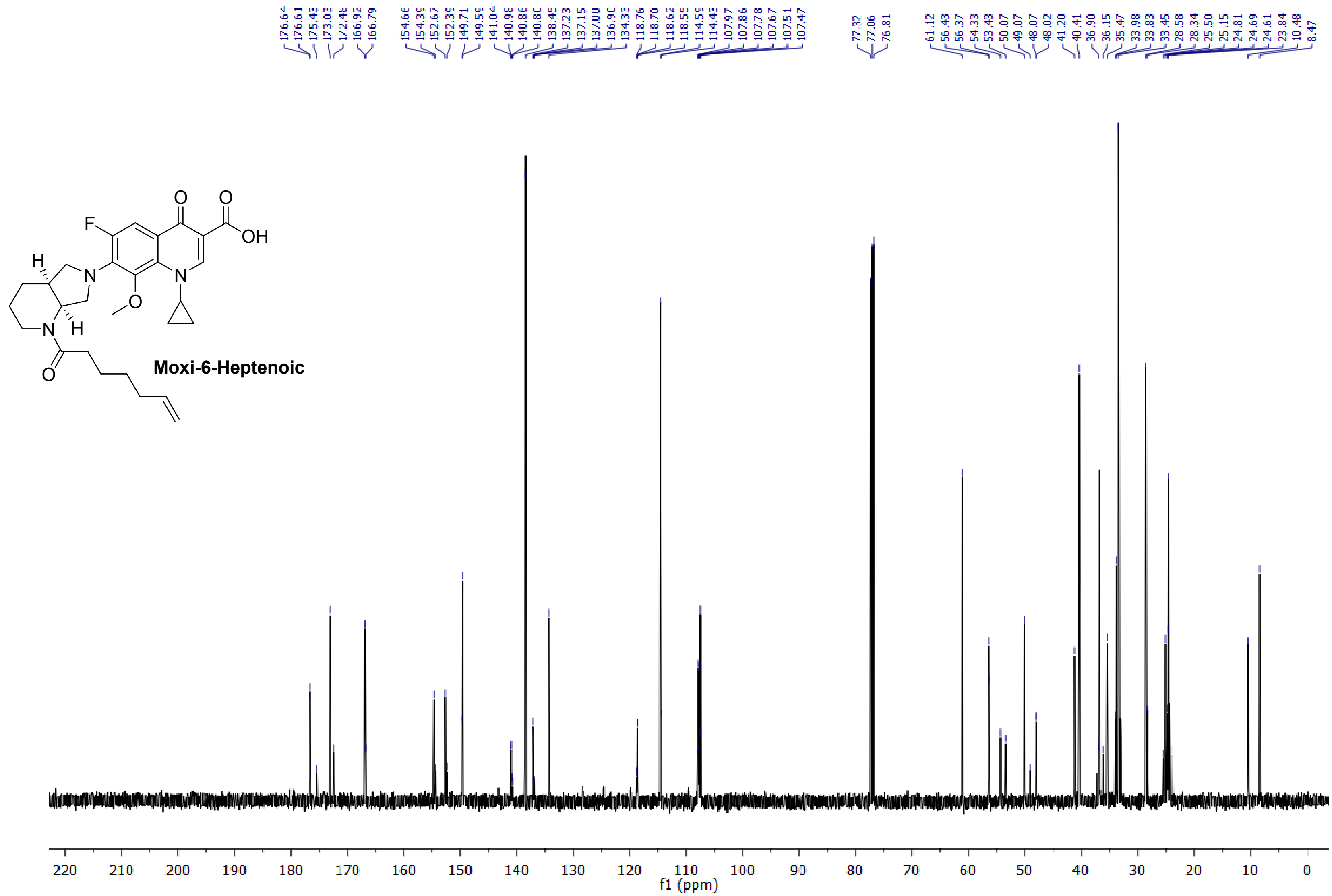

Moxi-6-Heptenoic

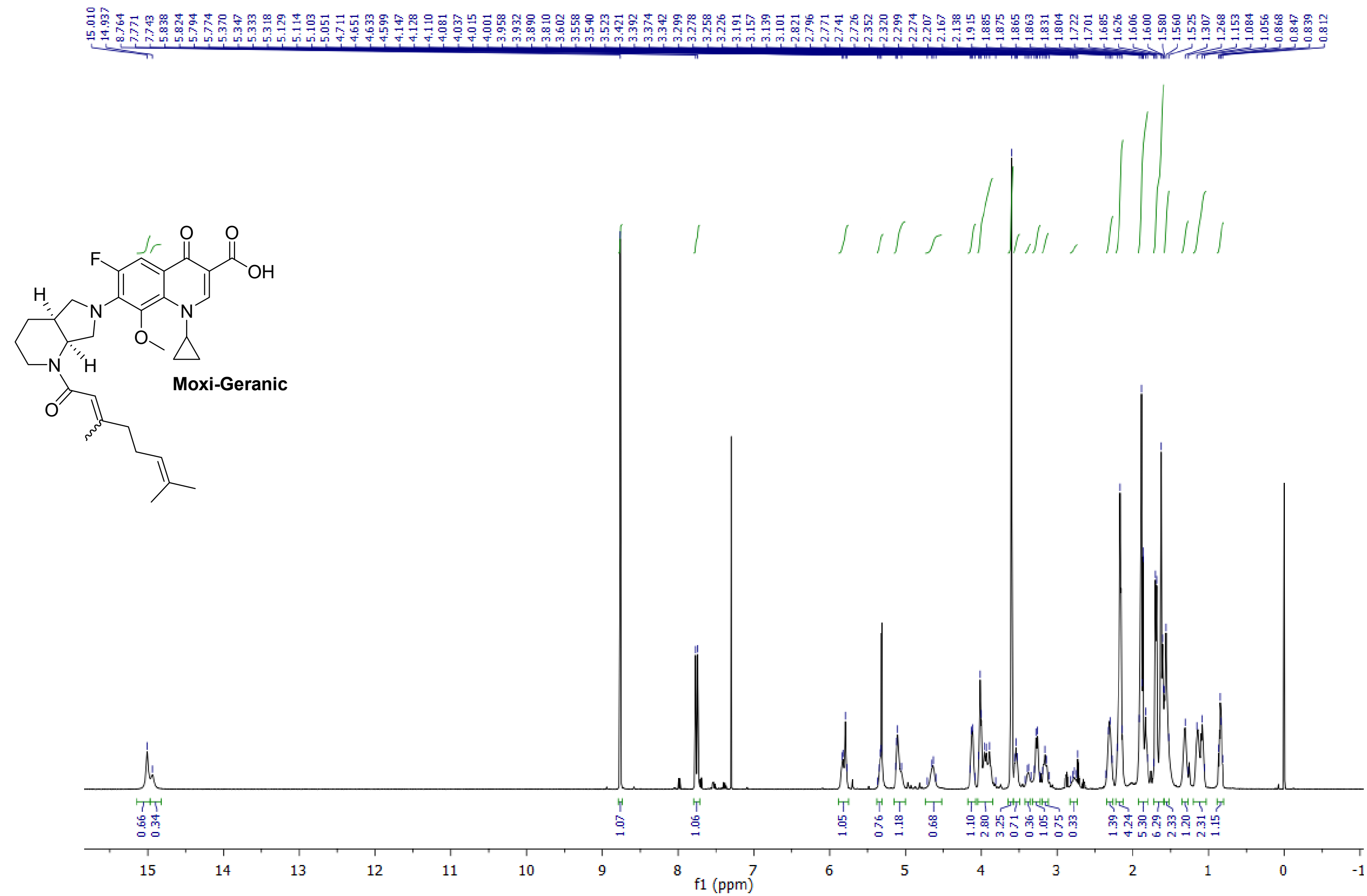
F
O
O
OH
N
N
H
H
N
O
O
Moxi-Geranic
f1 (ppm)

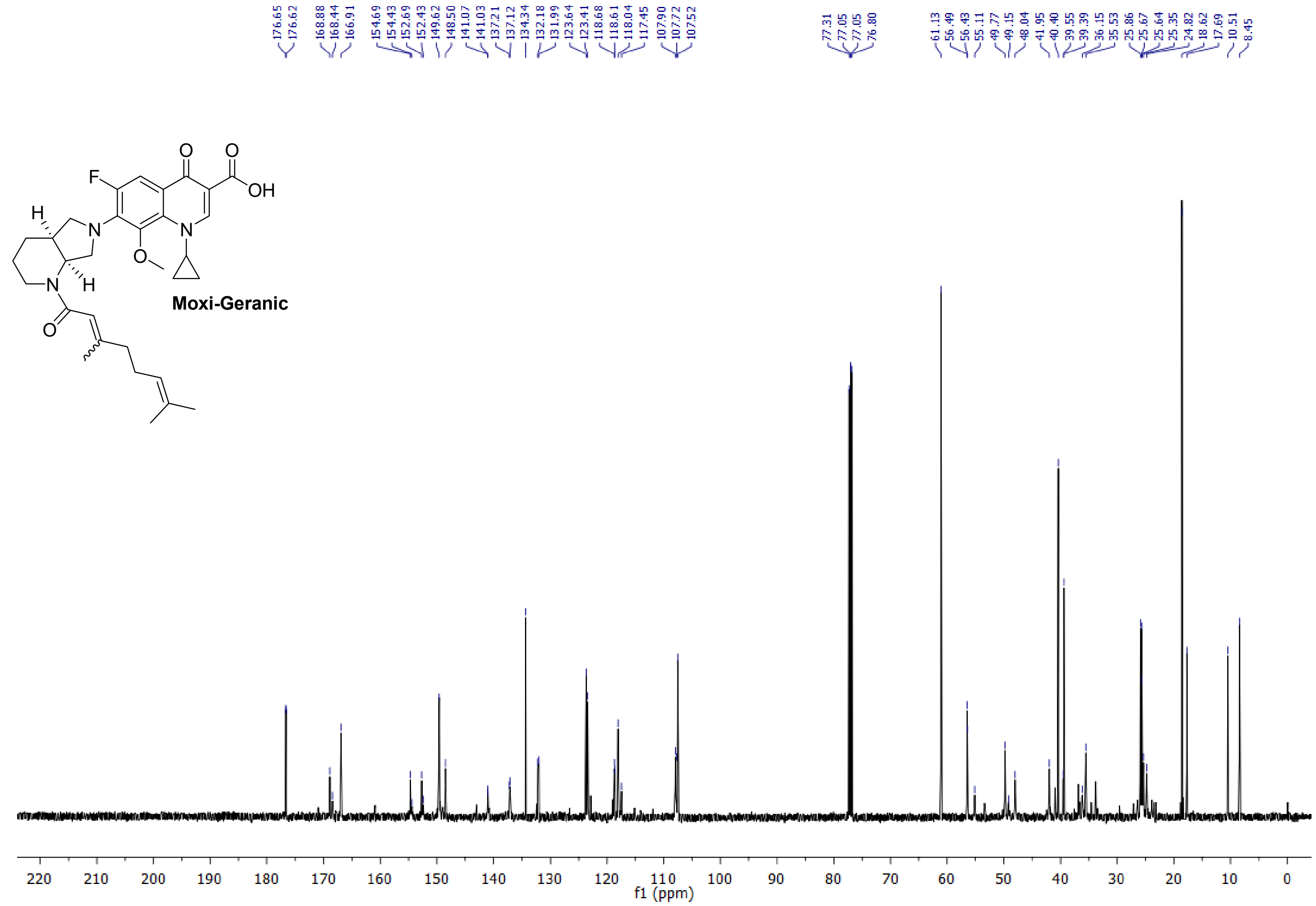
Moxi-Geranic
176.65
176.62
168.88
168.44
166.91
154.69
154.43
152.69
152.43
149.62
148.50
141.07
141.03
137.21
137.12
134.34
132.18
131.99
123.64
123.41
118.68
118.61
118.04
117.45
107.90
107.72
107.52
77.31
77.05
77.05
76.80
61.13
56.49
56.43
55.11
49.77
49.15
48.04
41.95
40.40
39.55
39.39
36.15
35.53
25.86
25.67
25.64
25.35
24.82
18.62
17.69
10.51
8.45
f1 (ppm)

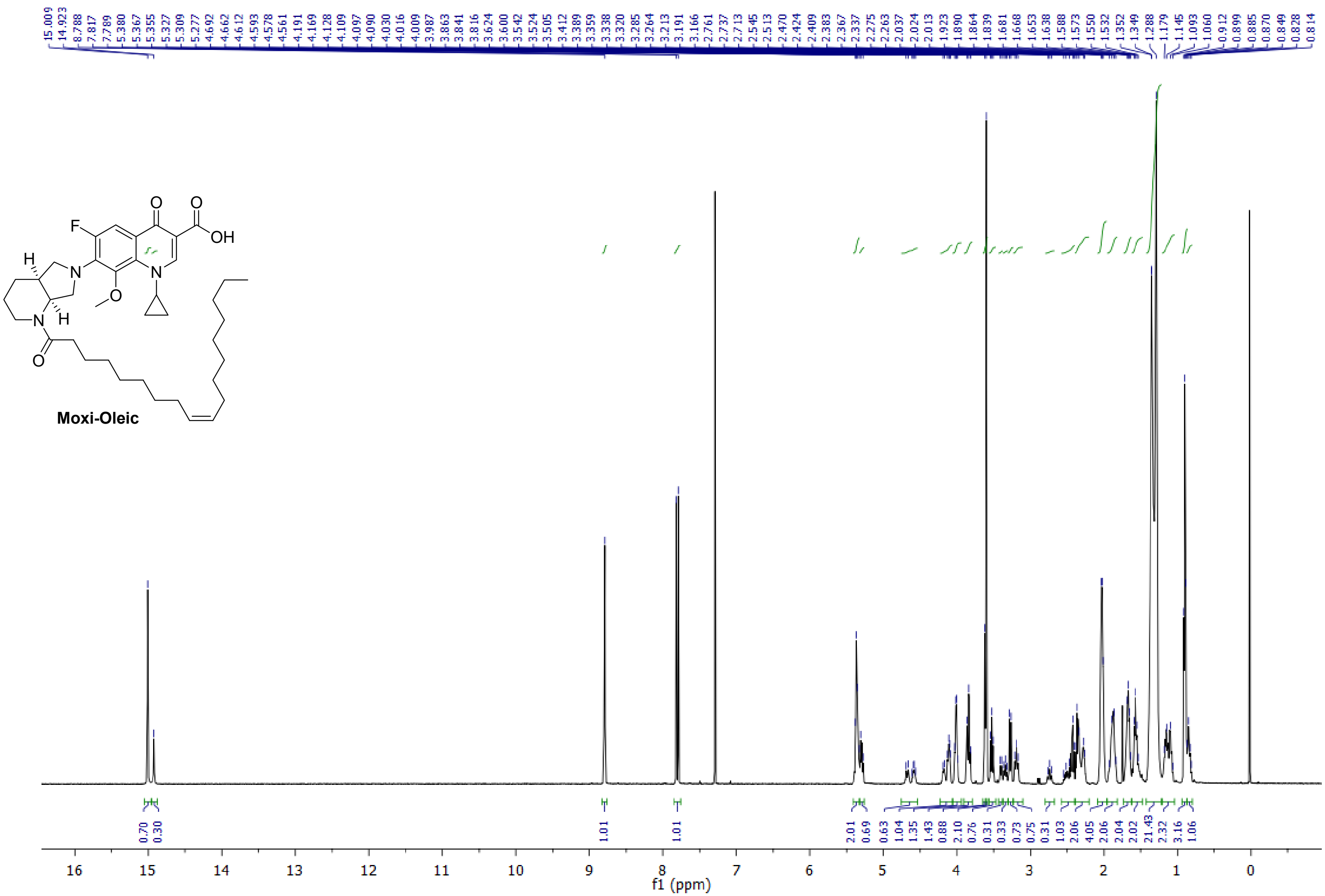
Moxi-Oleic
f1 (ppm)

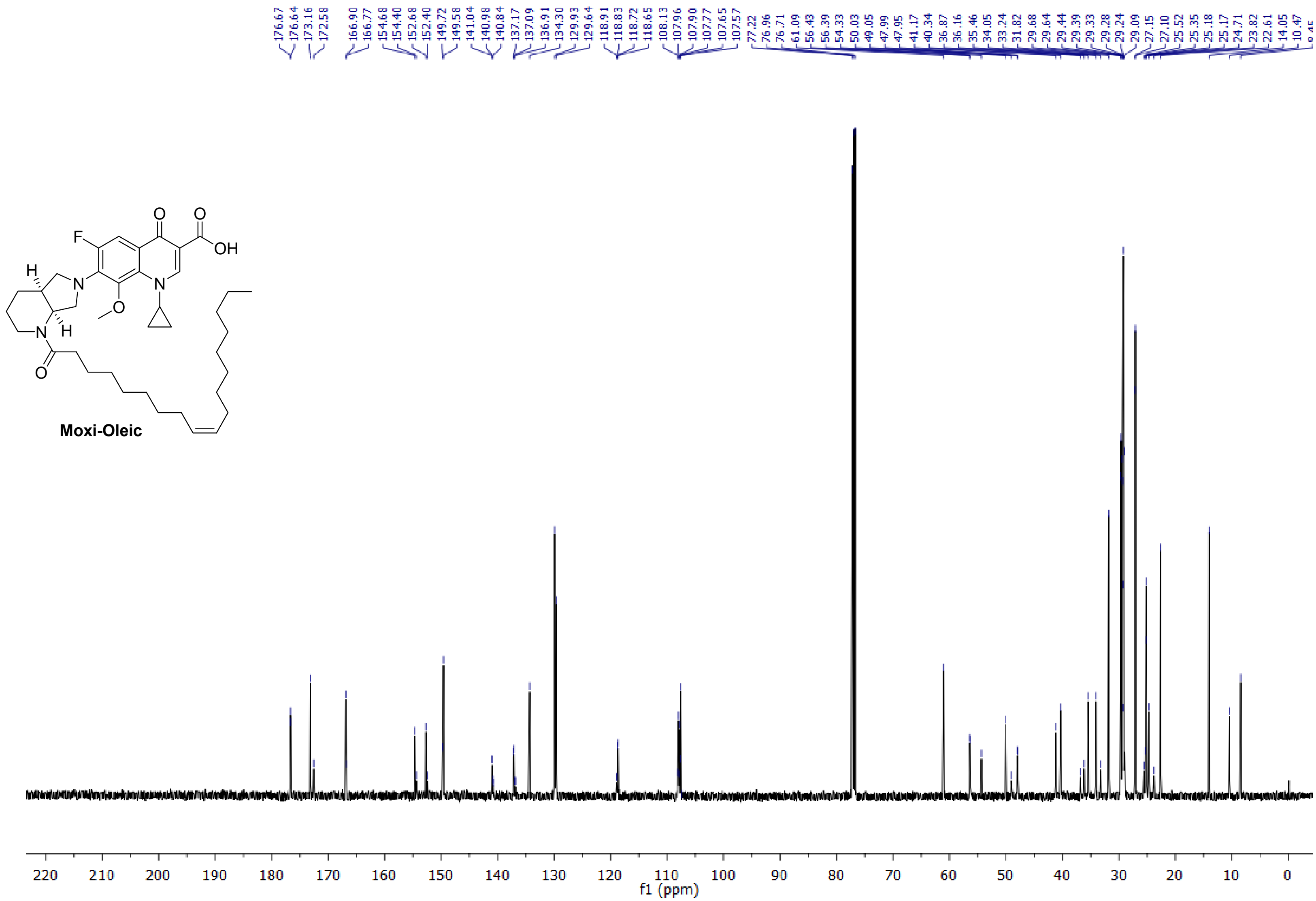

Moxi-Oleic
f1 (ppm)

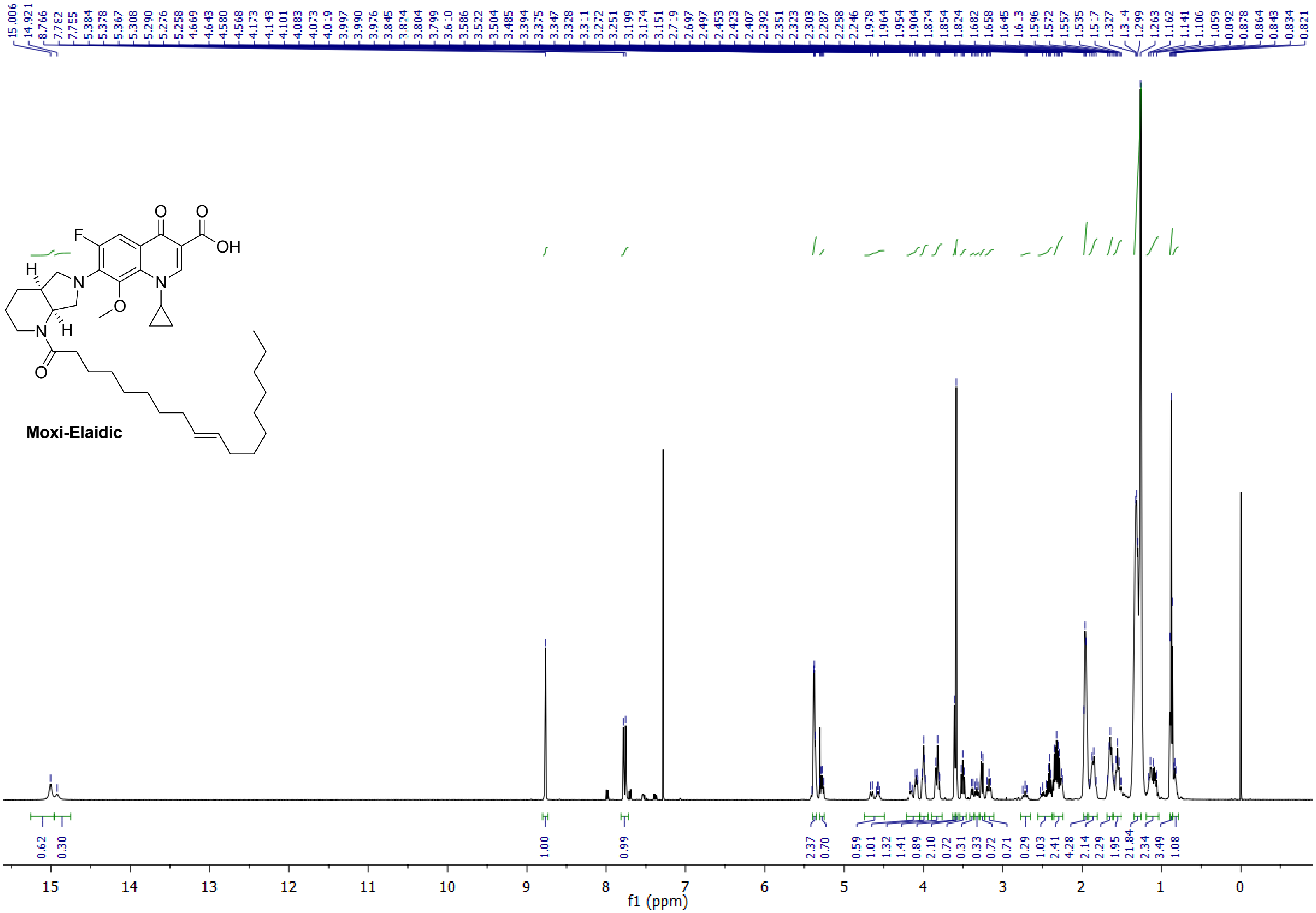
Moxi-Elaidic
f1 (ppm)

Moxi-Elaidic

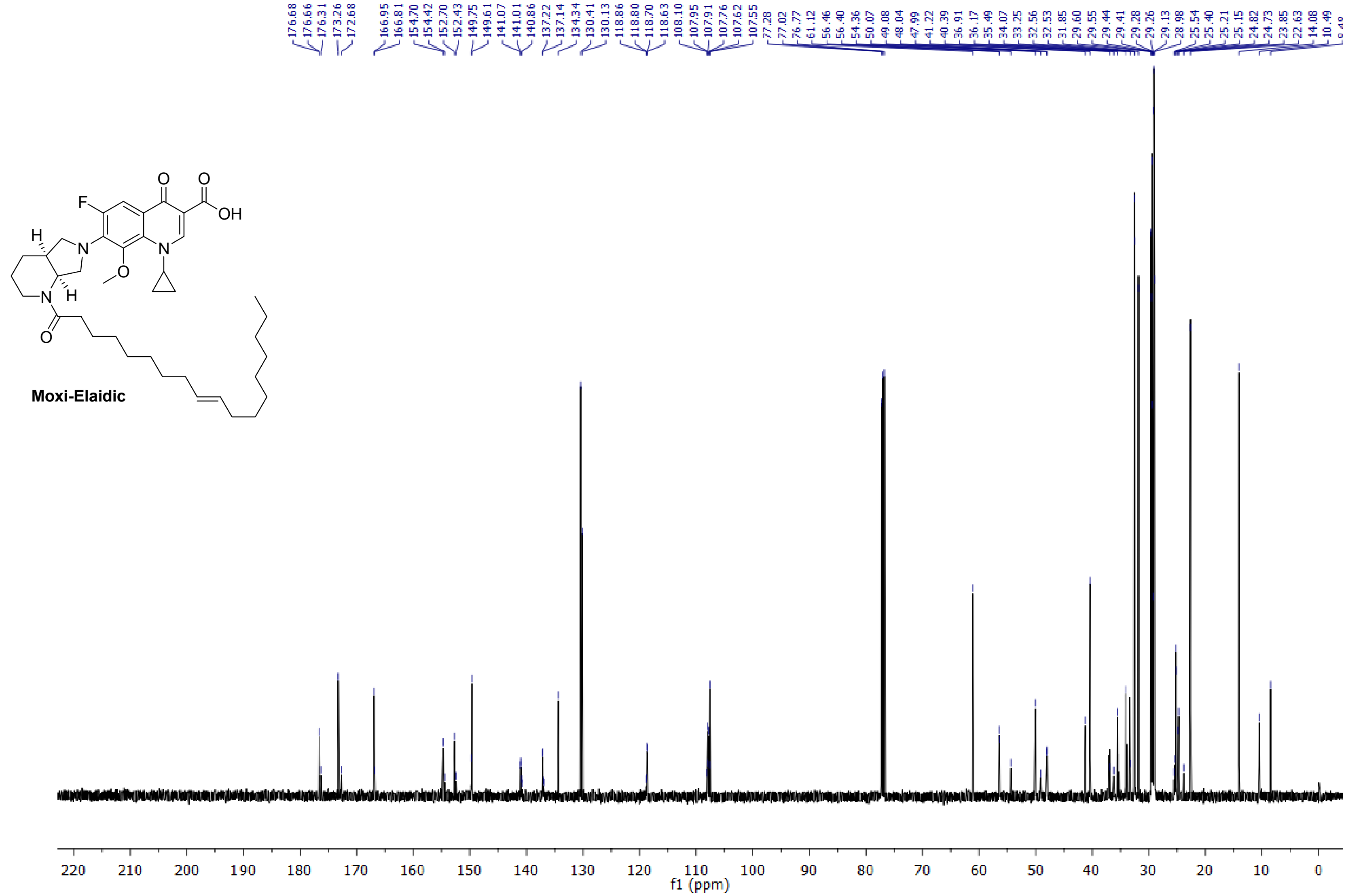

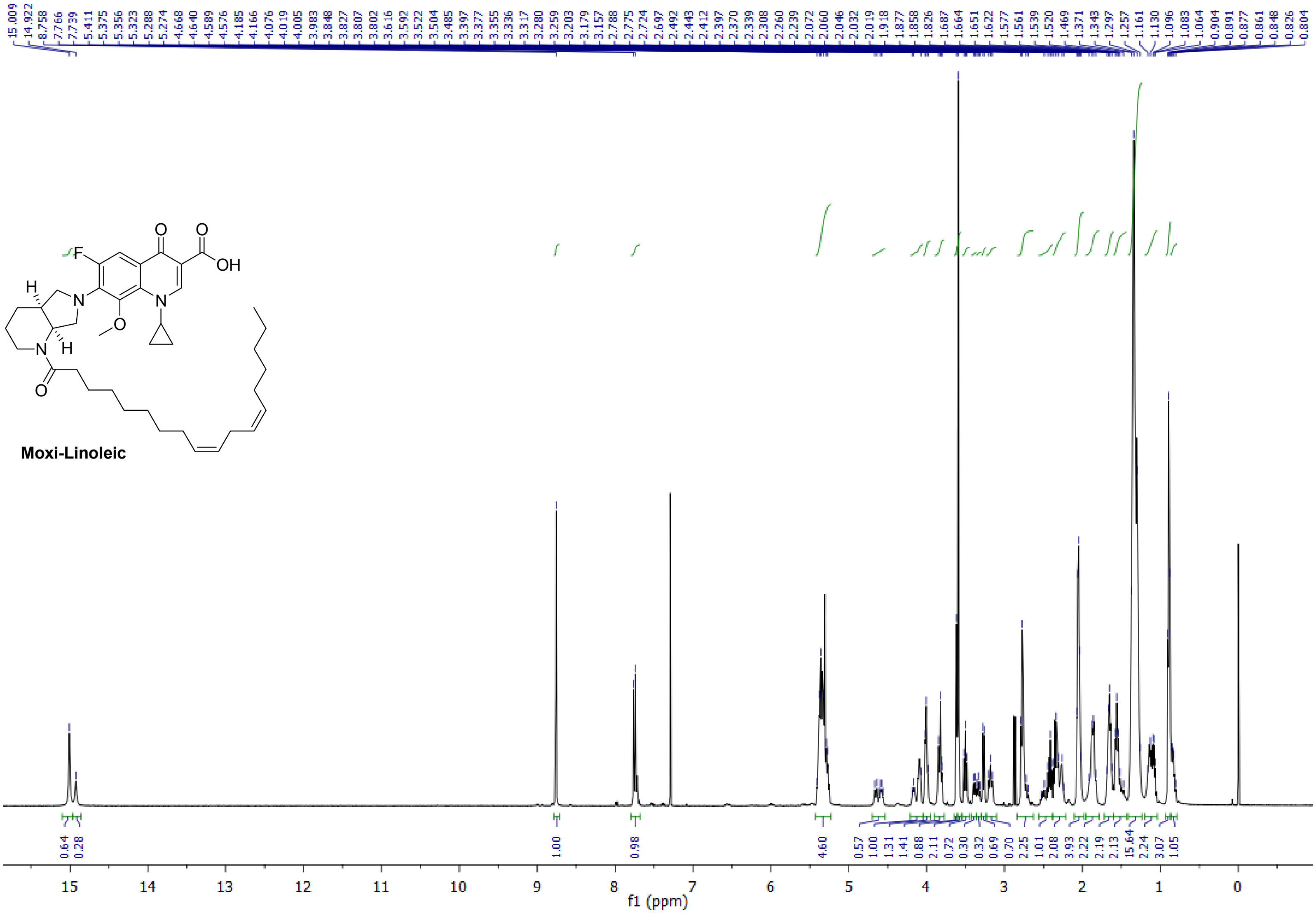

Moxi-Linoleic
f1 (ppm)

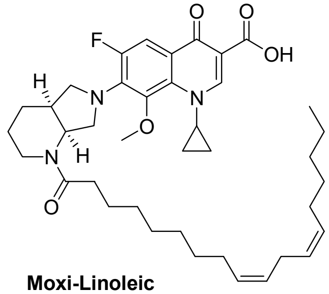

Moxi-Linoleic

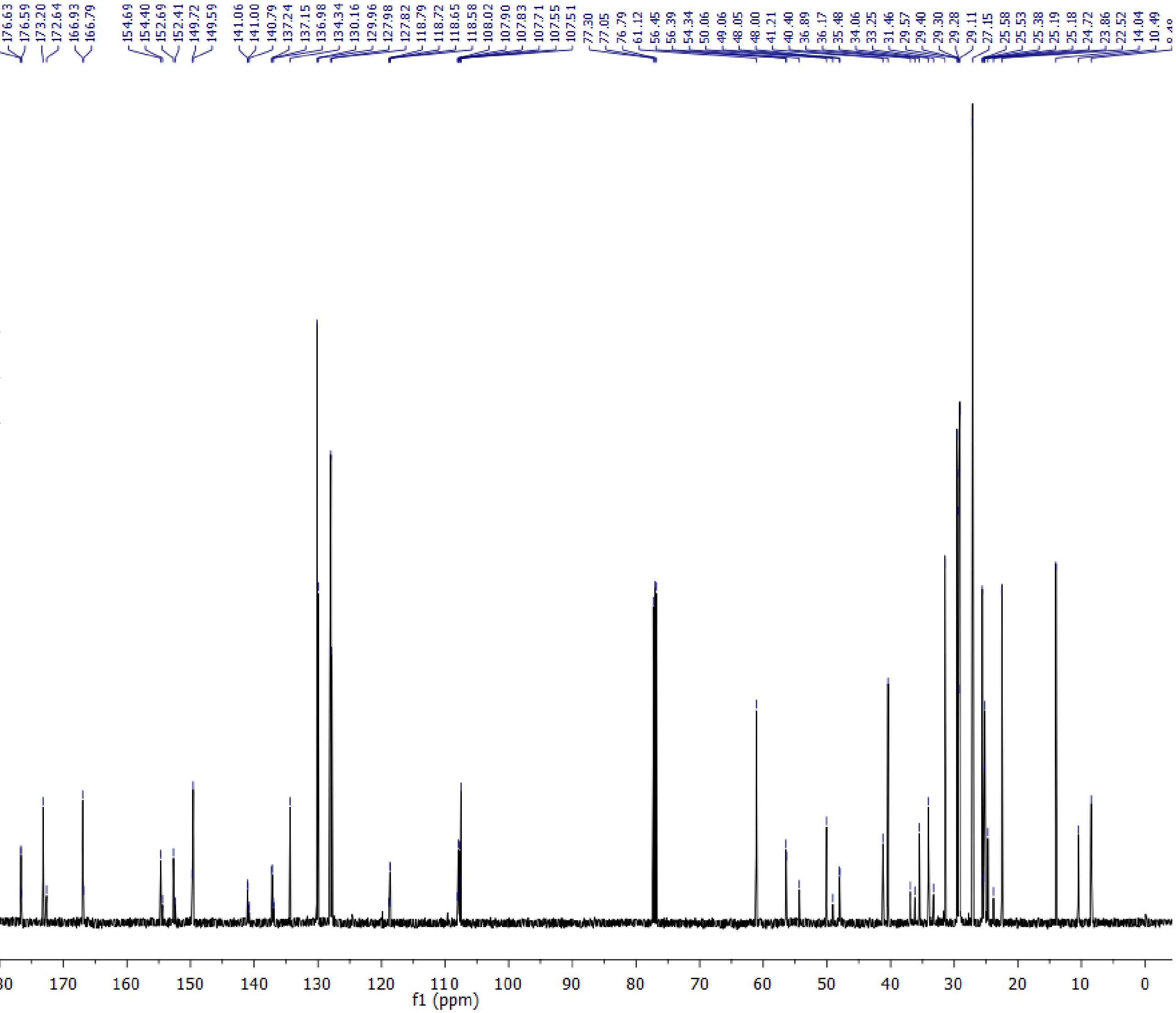

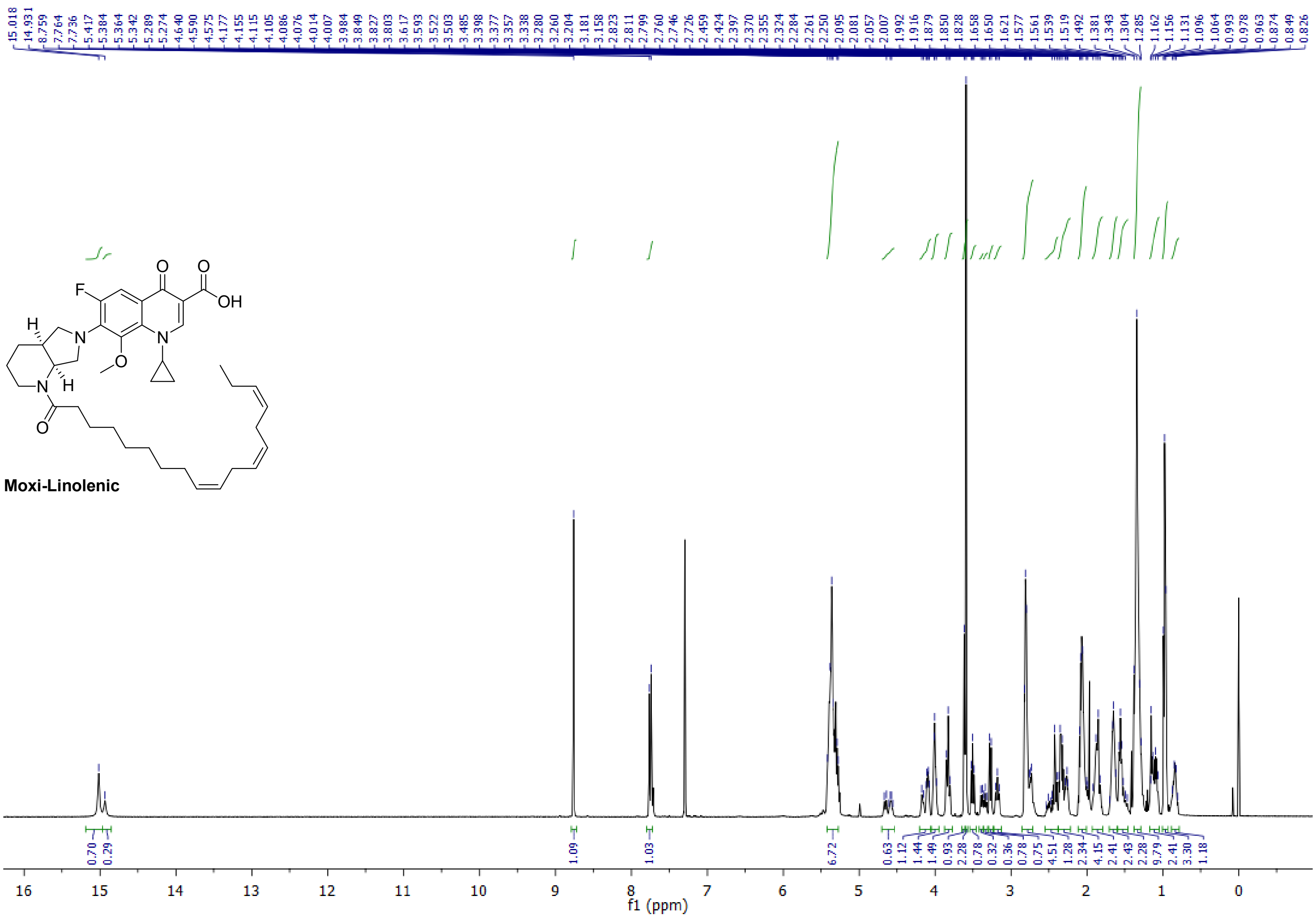

Moxi-Linolenic

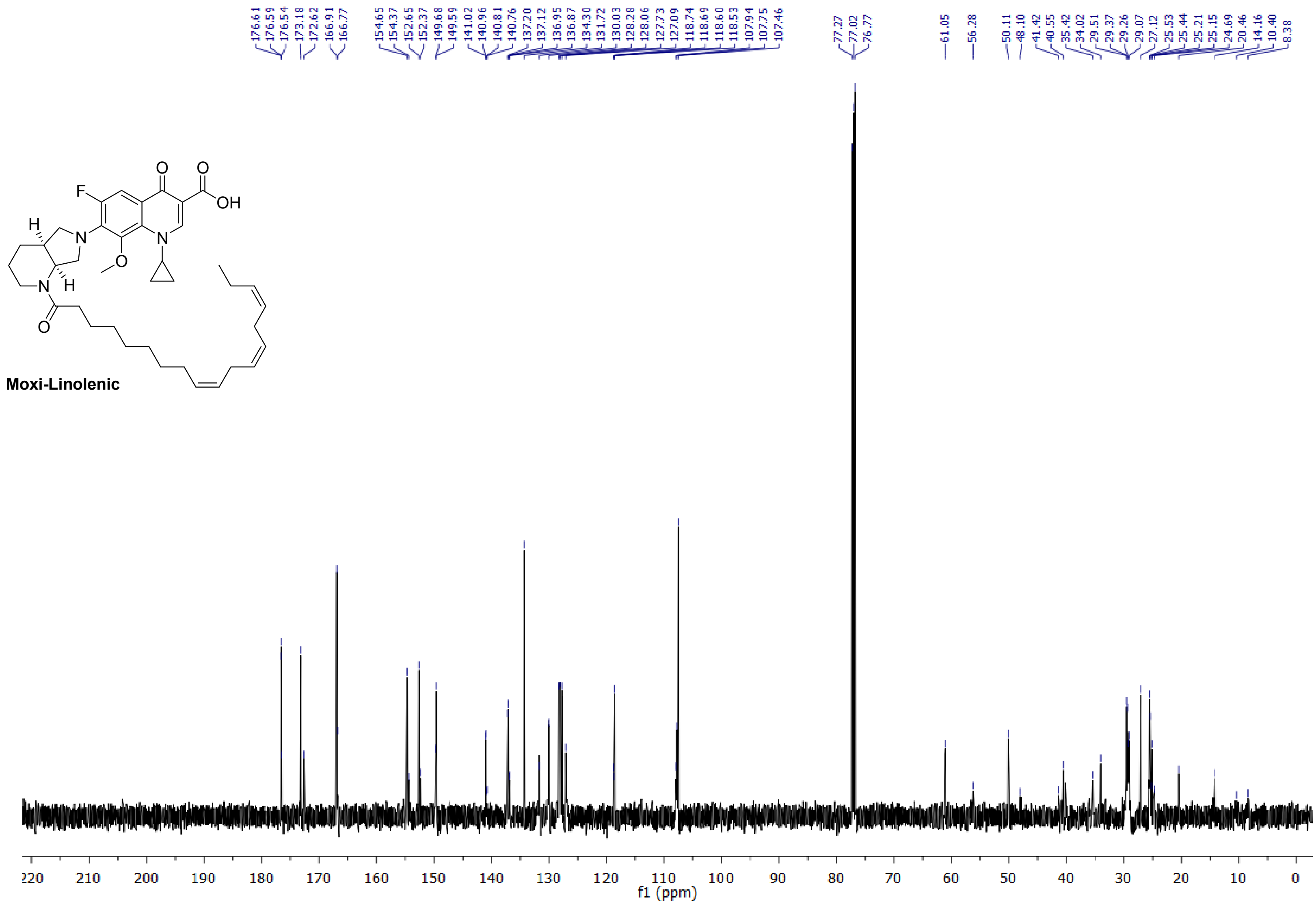
Moxi-Linolenic
176.61
176.59
176.54
173.18
172.62
166.91
166.77
154.65
154.37
152.65
152.37
149.68
149.59
141.02
140.96
140.81
140.76
137.20
137.12
136.95
136.87
134.30
131.72
130.03
128.28
128.06
127.73
127.09
118.74
118.69
118.60
118.53
107.94
107.75
107.46
77.27
77.02
76.77
61.05
56.28
50.11
48.10
41.42
40.55
35.42
34.02
29.51
29.37
29.26
29.07
27.12
25.53
25.44
25.21
25.15
24.69
20.46
14.16
10.40
8.38
f1 (ppm)

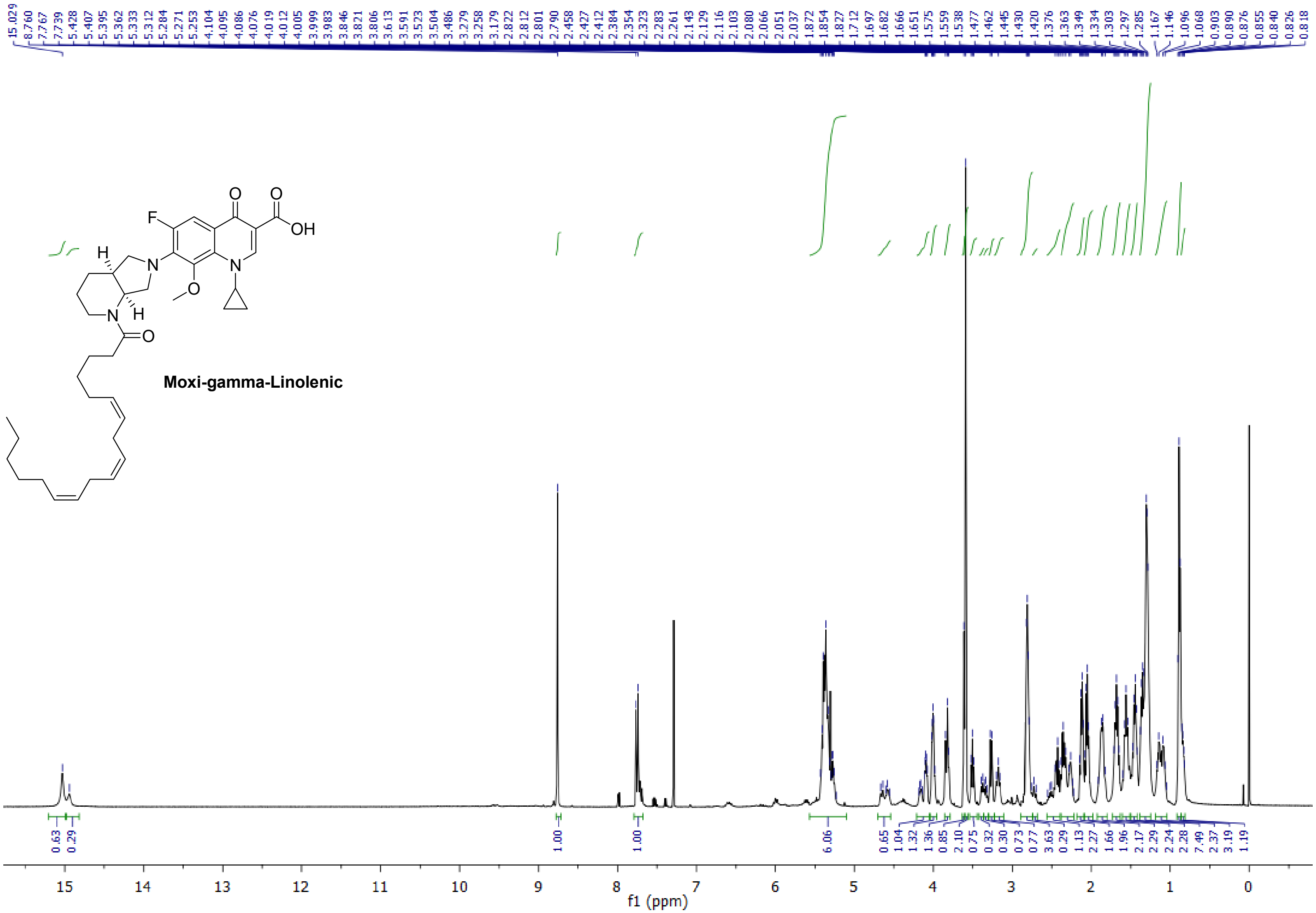

Moxi-gamma-Linolenic

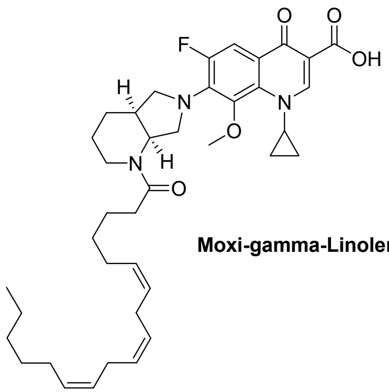

Moxi-gamma-Linolenic

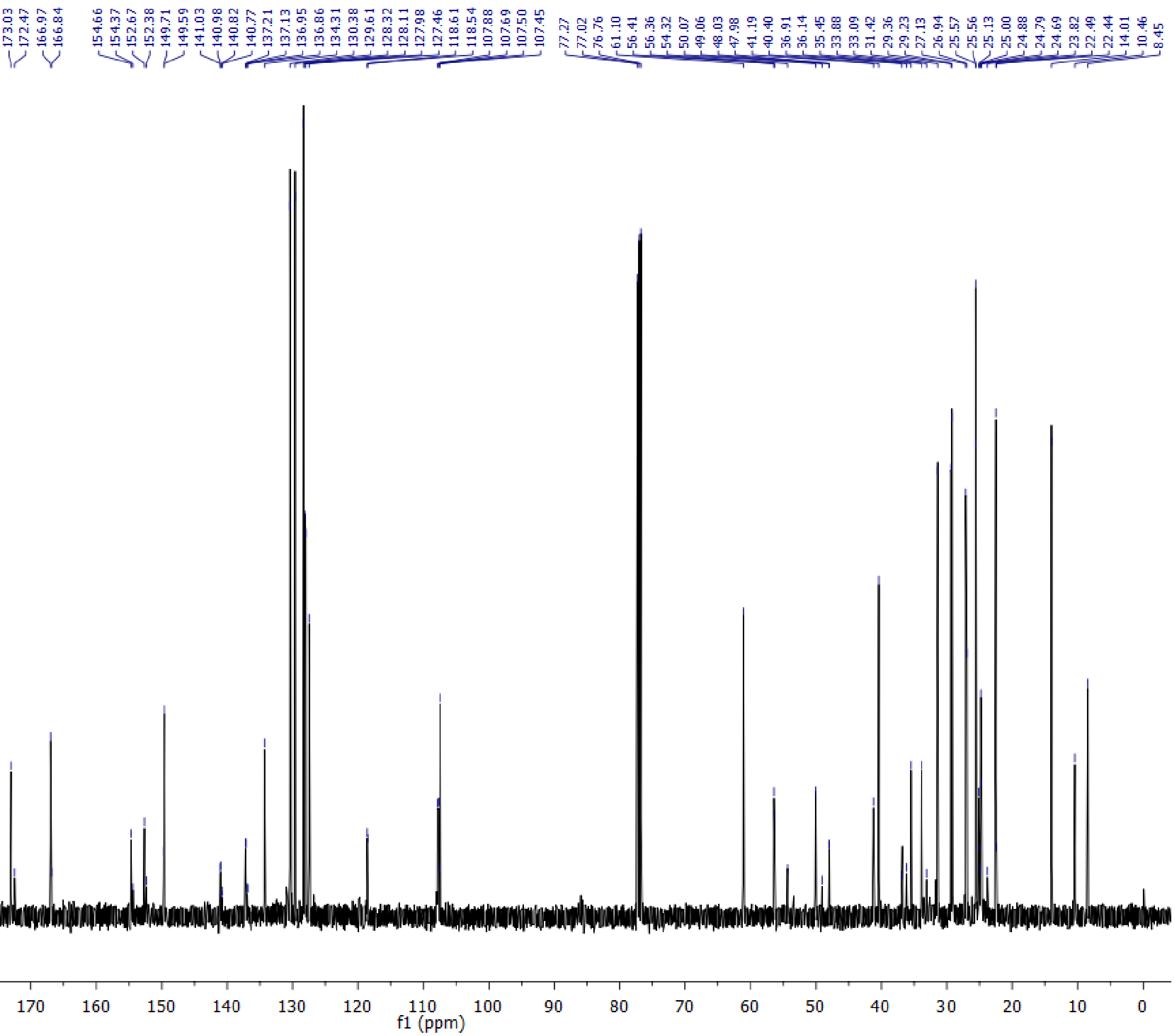

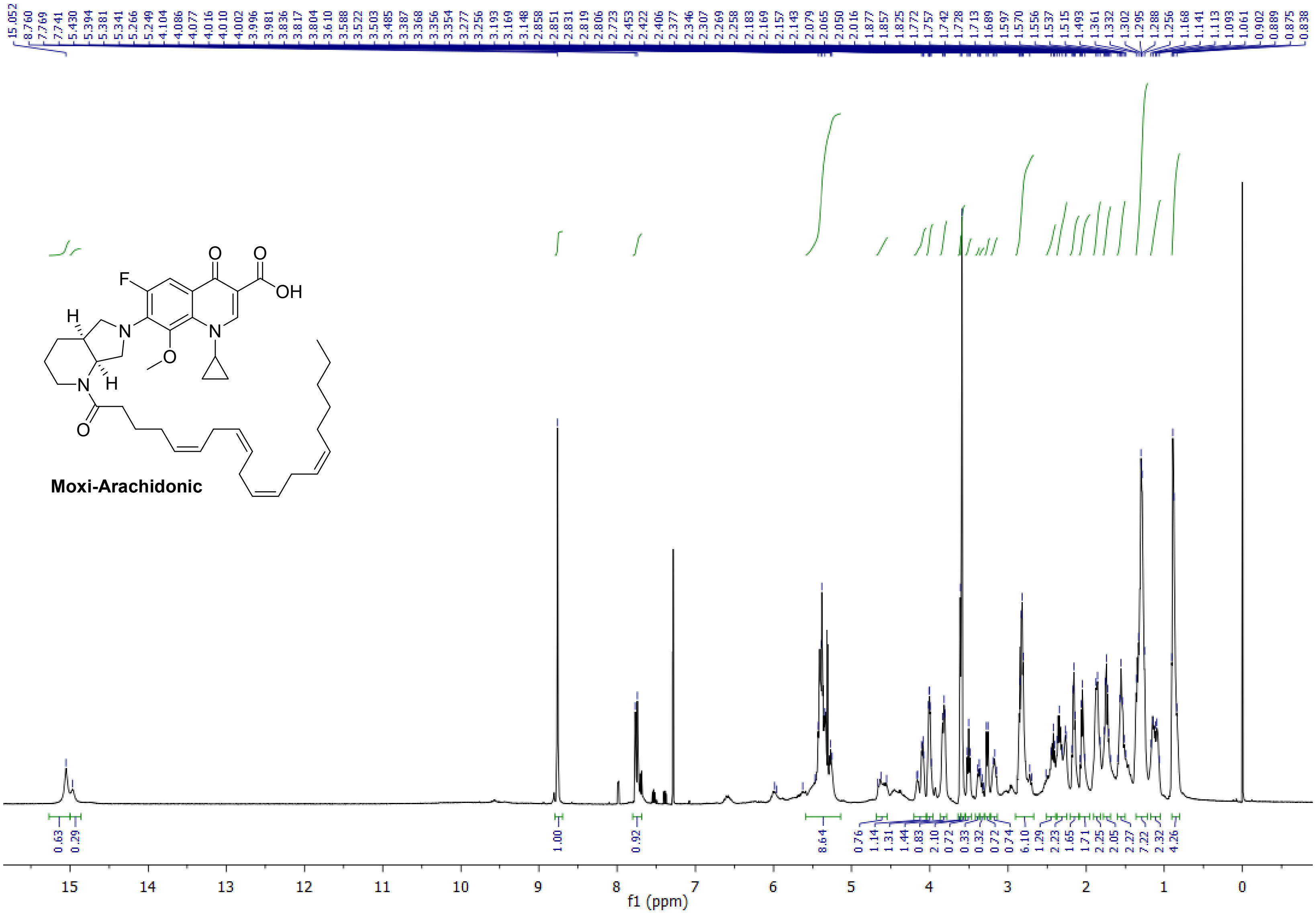

Moxi-Arachidonic
f1 (ppm)

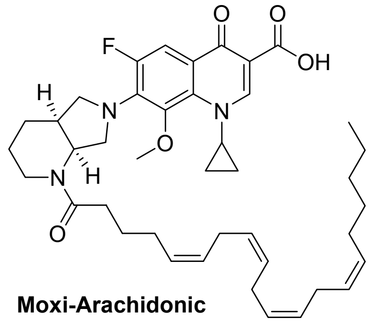

Moxi-Arachidonic

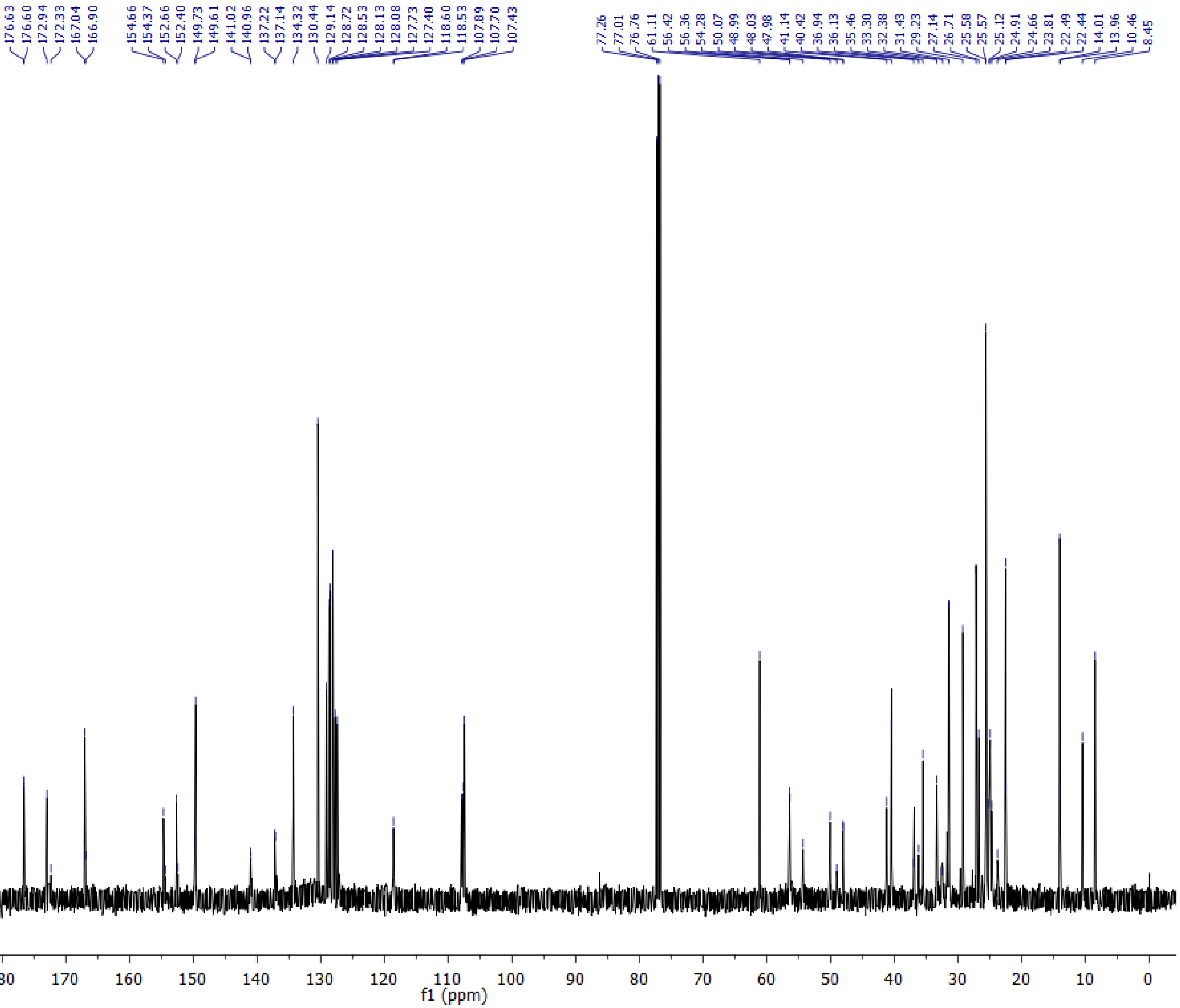

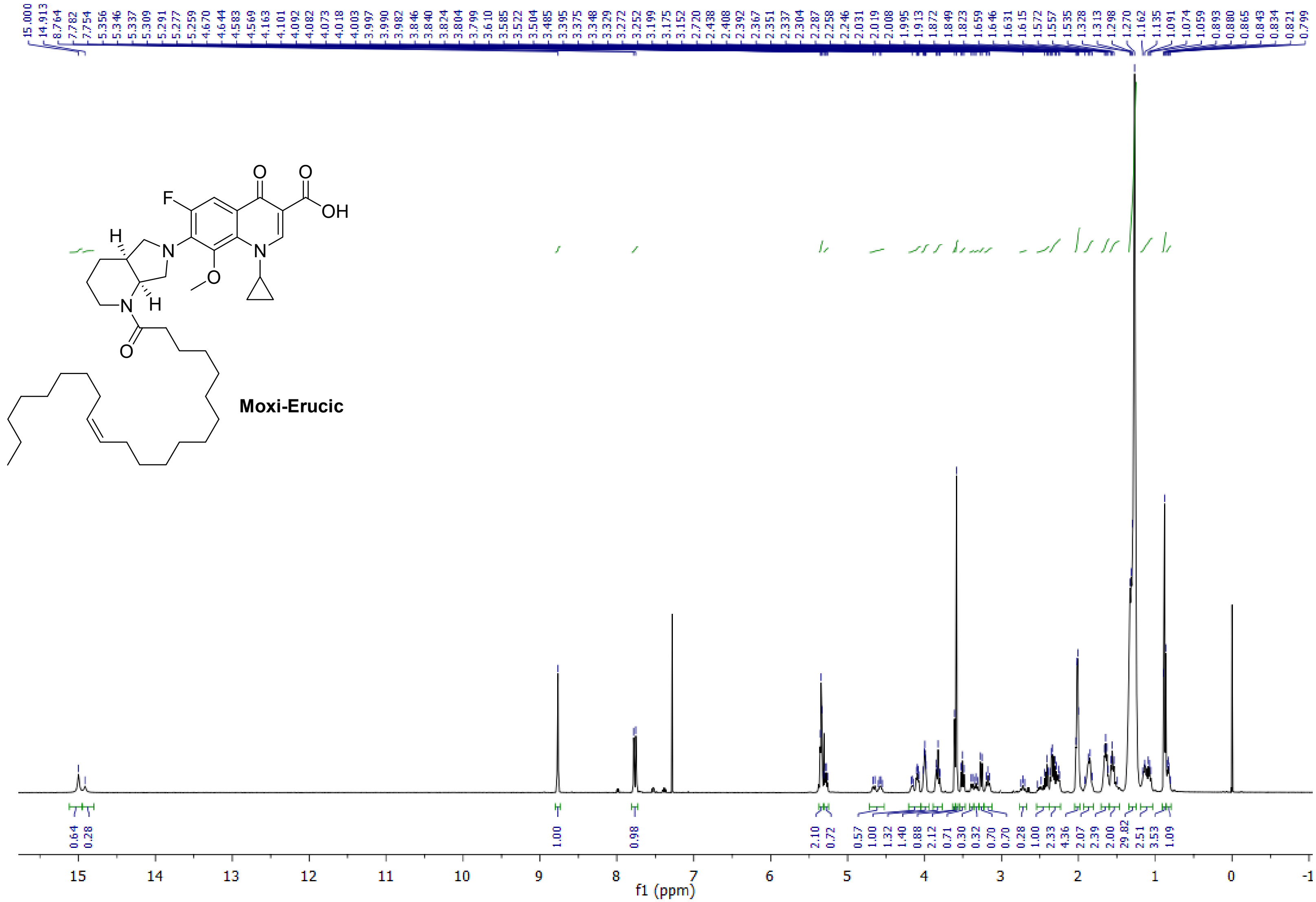

15.000
14.913
8.764
7.782
7.754
5.356
5.346
5.337
5.309
5.291
5.277
5.259
4.670
4.644
4.583
4.569
4.163
4.101
4.092
4.082
4.073
4.018
4.003
3.997
3.990
3.982
3.846
3.840
3.824
3.804
3.799
3.610
3.585
3.522
3.504
3.485
3.395
3.375
3.348
3.329
3.272
3.252
3.199
3.175
3.152
2.720
2.438
2.408
2.392
2.367
2.351
2.337
2.304
2.287
2.258
2.246
2.031
2.019
2.008
1.995
1.913
1.872
1.849
1.823
1.659
1.646
1.631
1.615
1.572
1.557
1.535
1.328
1.313
1.298
1.270
1.162
1.135
1.091
1.074
1.059
0.893
0.880
0.865
0.843
0.834
0.821
0.799
F
O
O
OH
H
N
N
O
N
H
O
Moxi-Erucic
0.64
0.28
1.00
0.98
2.10
0.72
0.57
1.00
1.32
1.40
0.88
2.12
0.71
0.30
0.32
0.70
0.70
0.28
1.00
2.33
4.36
2.07
2.39
2.00
29.82
2.51
3.53
1.09
15
14
13
12
11
10
9
8
7
6
5
4
3
2
1
0
-1
f1 (ppm)

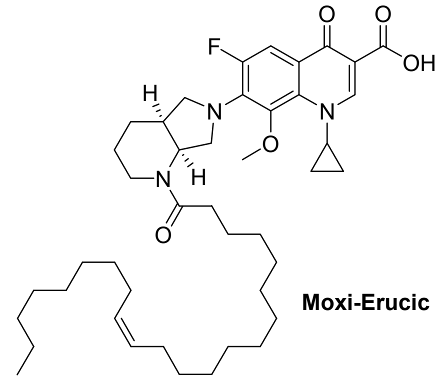
F
O
O
OH
H
N
N
O
H
N
O

Moxi-Erucic

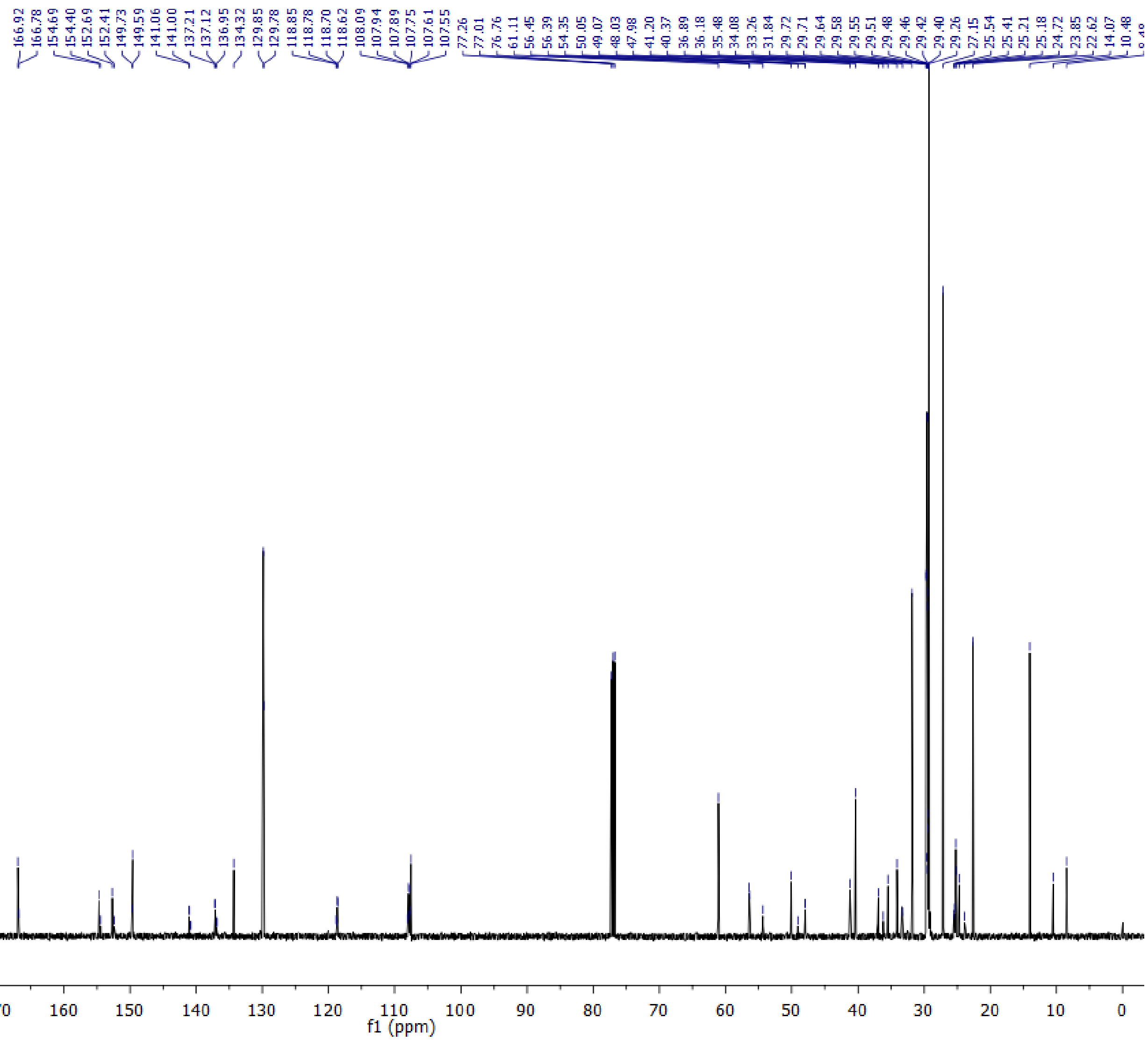
176.67
176.64
173.24
172.67
166.92
166.78
154.69
154.40
152.69
152.41
149.73
149.59
141.06
141.00
137.21
137.12
136.95
134.32
129.85
129.78
118.85
118.78
118.70
118.62
108.09
107.94
107.89
107.75
107.61
107.55
77.26
77.01
76.76
61.11
56.45
56.39
54.35
50.05
49.07
48.03
47.98
41.20
40.37
36.89
36.18
35.48
34.08
33.26
31.84
29.72
29.71
29.64
29.58
29.55
29.51
29.48
29.46
29.42
29.40
29.26
27.15
25.54
25.41
25.21
25.18
24.72
23.85
22.62
14.07
10.48
[illegible]
220 210 200 190 180 170 160 150 140 130 120 110 100 90 80 70 60 50 40 30 20 10 0
f1 (ppm)

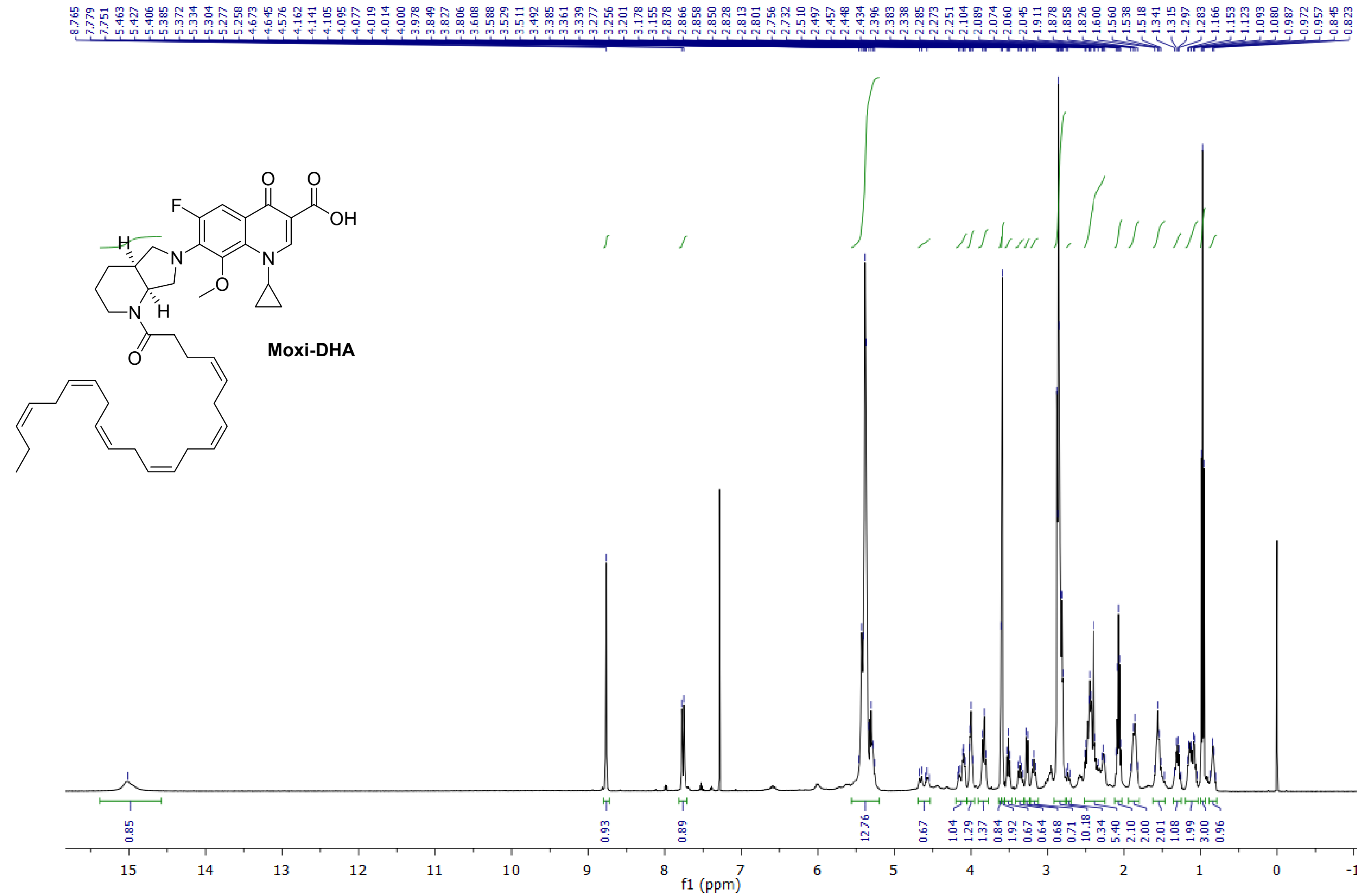
8.765
7.779
7.751
5.463
5.427
5.406
5.385
5.372
5.334
5.304
5.277
5.258
4.673
4.645
4.576
4.162
4.141
4.105
4.095
4.077
4.019
4.014
4.000
3.978
3.849
3.827
3.806
3.608
3.588
3.529
3.511
3.492
3.385
3.361
3.339
3.277
3.256
3.201
3.178
3.155
2.878
2.866
2.858
2.850
2.828
2.813
2.801
2.756
2.732
2.510
2.497
2.457
2.448
2.434
2.396
2.383
2.338
2.285
2.273
2.251
2.104
2.089
2.074
2.060
2.045
1.911
1.878
1.858
1.826
1.600
1.560
1.538
1.518
1.341
1.315
1.297
1.283
1.166
1.153
1.123
1.093
1.080
0.987
0.972
0.957
0.845
0.823
Moxi-DHA
0.85
0.93
0.89
12.76
0.67
1.04
1.29
1.37
0.84
1.92
0.67
0.64
0.68
0.71
10.18
0.34
5.40
2.10
2.00
2.01
1.08
1.99
3.00
0.96
15
14
13
12
11
10
9
8
7
6
5
4
3
2
1
0
-1
f1 (ppm)

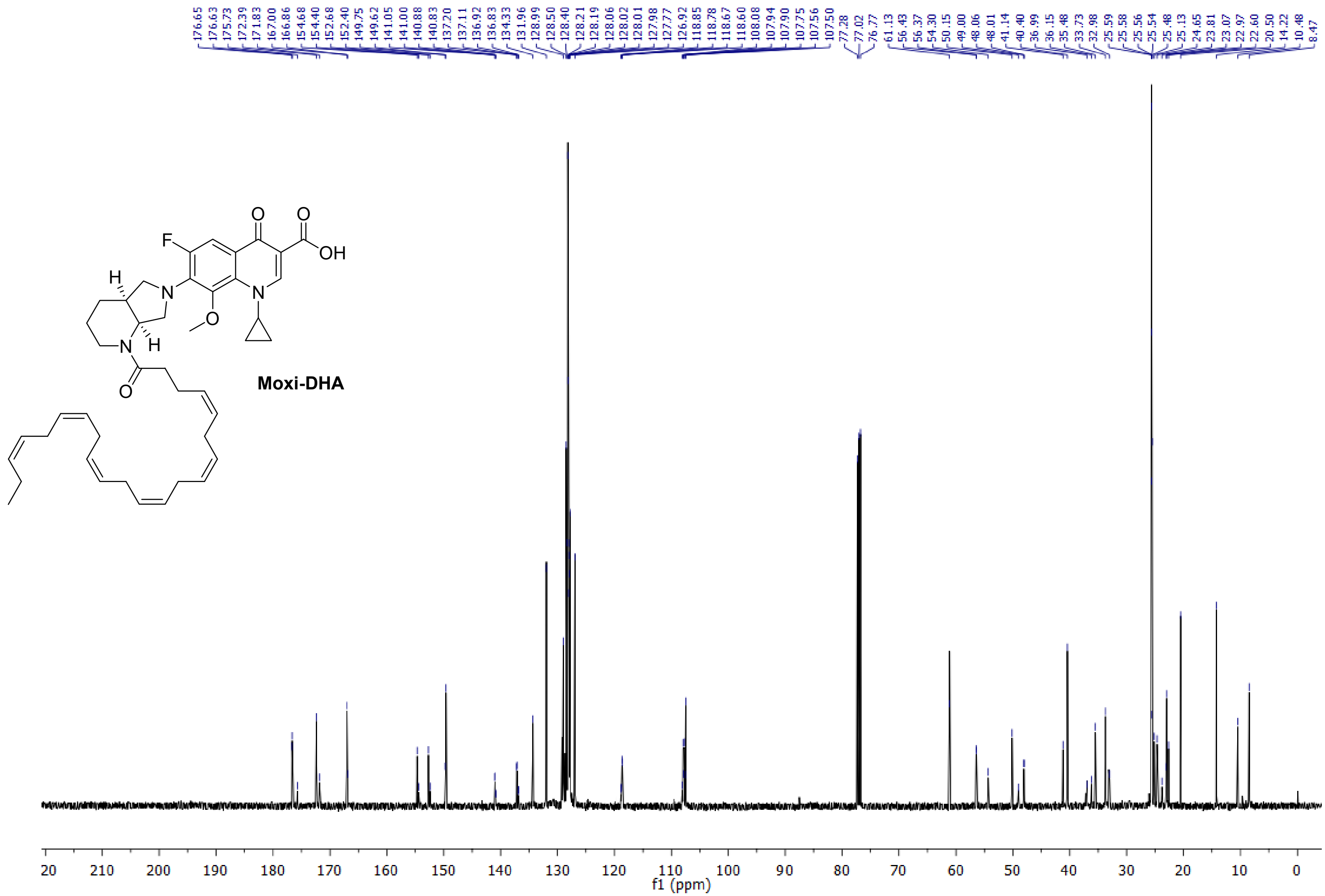

Moxi-DHA
F
O
O
OH
N
N
H
H
O
N
O
176.65
176.63
175.73
172.39
171.83
167.00
166.86
154.68
154.40
152.68
152.40
149.75
149.62
141.05
141.00
140.88
140.83
137.20
137.11
136.92
136.83
134.33
131.96
128.99
128.50
128.40
128.21
128.19
128.06
128.02
128.01
127.98
127.77
126.92
118.85
118.78
118.67
118.60
108.08
107.94
107.90
107.75
107.56
107.50
77.28
77.02
76.77
61.13
56.43
56.37
54.30
50.15
49.00
48.06
48.01
41.14
40.40
36.99
36.15
35.48
33.73
32.98
25.59
25.58
25.56
25.54
25.48
25.13
24.65
23.81
23.07
22.97
22.60
20.50
14.22
10.48
8.47
20
210
200
190
180
170
160
150
140
130
120
110
100
90
80
70
60
50
40
30
20
10
0
f1 (ppm)

Table S1. Evaluation of resistance phenotype of clinical strains used in assays.

	BETA-LACTAMS		AMINOGLYCOSIDES	QUINOLONES	MACROLIDES/ LINCOSAMIDES/ STREPTOGRAMINS	OXAZOLIDINONE	GLYCOPEPTIDES	TETRACYCLINES	RIFAMYCINES	TRIMETHOPRIM/ SULFONAMIDES
**KR 4047 825/19**	MR	MODIFICATION OF PBP (mecA)	RESISTANT KAN TOB GEN (APH(2")+AAC(6'))	RESISTANT, PARTIALLY RESISTANT	MLSB CONSTITUTIVE, MLSB+SA CONSTITUTIVE	WILD	RESISTANT (TEC)	TARGET MODIFICATION (TET M),PARTIALLY RESISTANT (efflux tet K)	WILD	TRIMETHOPRIM RESISTANT, WILD
**KR 4243 829/19**	MR	MODIFICATION OF PBP (mecA)	RESISTANT KAN (APH(3")-III), WILD RESISTENT KAN TOB (ANT(4')(4")	WILD	MLSB INDUCIBLE	WILD	WILD	WILD, PARTIALLY RESISTANT (efflux tet K)	WILD	TRIMETHOPRIM RESISTANT, WILD
**KR 4268 830/19**	MR	MODIFICATION OF PBP (mecA)	RESISTANT KAN TOB GEN (APH(2")+AAC(6'))	WILD	MLSB INDUCIBLE	WILD	WILD	WILD	WILD	TRIMETHOPRIM RESISTANT, WILD
**KR 4313 834/19**	MR	MODIFICATION OF PBP (mecA)	RESISTANT KAN (APH(3")-III), WILD RESISTENT KAN TOB (ANT(4')(4")	WILD	MLSB CONSTITUTIVE, MLSB+SA CONSTITUTIVE	WILD	RESISTANT (TEC)	WILD, PARTIALLY RESISTANT (efflux tet K)	WILD	TRIMETHOPRIM RESISTANT, WILD
**KR 4358/2 840/19**	MR	MODIFICATION OF PBP (mecA)	RESISTANT KAN (APH(3")-III), WILD RESISTENT KAN TOB (ANT(4')(4")	WILD	WILD	WILD	WILD	WILD	WILD	RESISTANT
**T 5253 845/19**	MR	MODIFICATION OF PBP (mecA)	RESISTANT KAN TOB GEN (APH(2")+AAC(6'))	RESISTANT, PARTIALLY RESISTANT	WILD	WILD	WILD	WILD, PARTIALLY RESISTANT (efflux tet K)	WILD	RESISTANT
**T 5399 848/19**	MR	MODIFICATION OF PBP (mecA)	RESISTANT KAN (APH(3")-III), WILD RESISTENT KAN TOB (ANT(4')(4")	WILD	WILD	WILD	WILD	WILD, PARTIALLY RESISTANT (efflux tet K)	WILD	TRIMETHOPRIM RESISTANT, WILD
**T 5501 851/19**	MR	MODIFICATION OF PBP (mecA)	RESISTANT KAN TOB GEN (APH(2")+AAC(6'))	WILD	MLSB INDUCIBLE	WILD	WILD	WILD, PARTIALLY RESISTANT (efflux tet K)	WILD	TRIMETHOPRIM RESISTANT, WILD
